# An olfactory virtual reality system for mice

**DOI:** 10.1038/s41467-018-03262-4

**Published:** 2018-02-26

**Authors:** Brad A. Radvansky, Daniel A. Dombeck

**Affiliations:** Department of Neurobiology, Northwestern University, Evanston, IL 60208 USA

## Abstract

All motile organisms use spatially distributed chemical features of their surroundings to guide their behaviors, but the neural mechanisms underlying such behaviors in mammals have been difficult to study, largely due to the technical challenges of controlling chemical concentrations in space and time during behavioral experiments. To overcome these challenges, we introduce a system to control and maintain an olfactory virtual landscape. This system uses rapid flow controllers and an online predictive algorithm to deliver precise odorant distributions to head-fixed mice as they explore a virtual environment. We establish an odor-guided virtual navigation behavior that engages hippocampal CA1 “place cells” that exhibit similar properties to those previously reported for real and visual virtual environments, demonstrating that navigation based on different sensory modalities recruits a similar cognitive map. This method opens new possibilities for studying the neural mechanisms of olfactory-driven behaviors, multisensory integration, innate valence, and low-dimensional sensory-spatial processing.

## Abstract (web summary)

Odor-guided spatial behaviours are difficult to study due to the challenge of controlling chemical concentrations in space and time. Here the authors present a precise odor delivery system to generate a olfactory virtual landscape that engages hippocampal place cells in mice.

## Introduction

Virtual reality (VR) offers unique experimental capabilities for studying the neural basis of animal behavior^1,2^. This technology provides precise control over the animal’s sensory environment, and therefore can be used to establish relationships between sensory features of an environment and the tuning properties of individual neurons that can be difficult to discern in real-world conditions^3,4^. It enables experiments that are either not possible or difficult to realize in real environments, such as generating cue conflict stimuli, delivering non-natural sudden changes in stimuli at particular locations, and observing sensory-driven behavior in isolation from other sensory information, such as walls/borders, textures, self-generated odors, and vestibular cues. Importantly, behaving animals can be head-restrained in VR, allowing for the application of advanced brain recording and stimulating techniques, such as whole-cell patch clamping^5–7^, two-photon imaging^8–14^, and two-photon stimulation^15,16^ that can reveal circuit^15–17^, cellular^5–7^, and sub-cellular^11^ mechanisms underlying behavior.

The vast majority of VR studies to date have used visually defined environments^3–9,11,14–20^. Tactile^21^ and auditory^22^ VR systems have also been established. Yet, despite the importance of odor-driven behaviors for mammals, few attempts have been made to fully incorporate odor into VR. Olfactory cues have been delivered to a mouse via airflow in an on/off manner during experience in different visual^23^ or multisensory^10,12^ VR environments in order to create a contextual association with other stimuli. Additionally, creative use of odor trails drawn on a treadmill has been validated for studying odor tracking in rats^24^.

Yet, a method to control odorant concentration as a continuous function of virtual space does not currently exist. State-of-the-art VR olfactometers (PhenoSys) operate with a delay of 0.5–1 s, which is too slow to provide a reproducible and high-resolution odorant spatial distribution for rodents that run at variable speeds on the order of ~0.5 m s^[−15,]^^19^, sniff at rates of 3–12 Hz^25^ and can perform odor-driven behaviors on the order of ~100 ms^25,26^. Millisecond-timescale olfactometers have been designed for delivering odorant puffs^26–28^, but have not been validated for controlling concentration as a continuous variable for long durations, and have not been incorporated into VR. Because of these limitations, mammalian VR experiments have been restricted to using odor as a categorical variable. While this approach has allowed for studying high-level cognitive processes, such as associational memory, it is insufficient for addressing more elementary questions of how the brain can represent and generate behaviors within a continuous olfactory world. To address such questions, here we establish an olfactory VR system capable of controlling odors as continuous spatial variables. To validate this system for behavioral and neural applications for head-fixed mice, we establish an olfactory virtual navigation behavior that engages hippocampal “place cells.” This demonstrates that an environment comprised of only olfactory features, combined with self-motion cues, can engage hippocampal cognitive mapping mechanisms.

## Results

### Rapid, consistent odorant delivery in VR

**Fig. 1:**
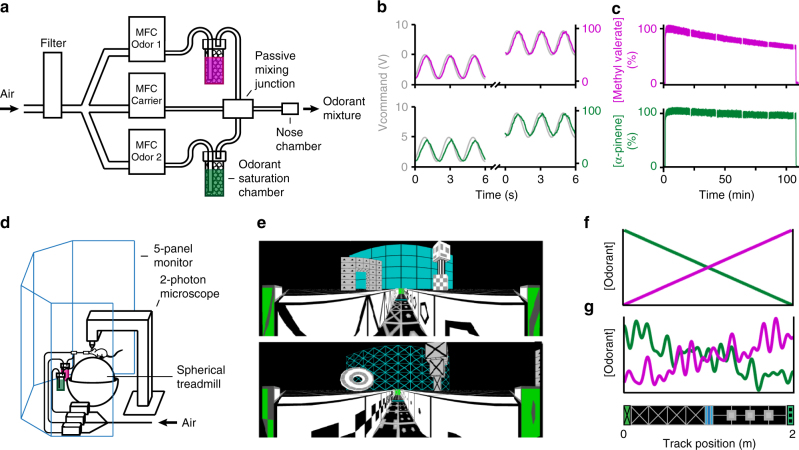
Odorant delivery in VR. **a** Two mass flow controllers (MFCs) in parallel sent odorants to the nose. The odorants were methyl valerate (bubblegum smell, pink) and α-pinene (pine smell, green). A third MFC sending blank air was dynamically updated to maintain a constant final flow rate (1 L min^−1^). The three streams converged at a passive mixing block and flowed to a nose chamber that covered the snout of the mouse (Supplementary Fig. 1). **b** Rapid, continuous odorant delivery following a 0.5-Hz sinusoidal command of low and high offset. Relative odorant concentration was measured using a photo-ionization detector (PID) placed at the nose chamber. **c** Stable odorant delivery at maximum flow rate (0.1 L min^−1^) for ~100 min measured with PID at nose chamber. Small gaps occur where baseline measurements were taken to calibrate the PID (Supplementary Fig. 2). **d** The odorant delivery apparatus incorporated into an existing visual VR system in which a head-fixed mouse ran on a spherical treadmill facing a 5-panel monitor beneath a 2-photon microscope. **e** Top: view from the α-pinene side of the track. Bottom: view from the methyl valerate side of the track. **f** Idealized smooth odorant spatial distributions. **g** Idealized example noisy odorant spatial distributions (top). Cartoon of the visual virtual track (bottom)

To control a continuous odorant distribution across virtual space requires rapid odorant delivery/clearance relative to the timescale of the subject’s movement^5^ (~0.5 m s^−1^) and sniff cycle^25^ (3–12 Hz). Further, to maintain this distribution for the duration of a behavioral session requires consistent odorant delivery with minimal depletion over time (~30 min). To achieve these criteria, we developed an olfactometer comprised of the fastest available mass flow controllers (MFCs), the smallest tube/bottle volumes possible without compromising airflow, and a novel rapid odorant saturation chamber (Fig. 1a) (Methods section). This olfactometer controlled two independent odorant streams (flow rate 0.001–0.1 L min^−1^ passed through rapid odorant saturation chambers) that met a third carrier stream (flow rate 0.8–1 L min^−1^, containing blank air) at a passive mixing block leading to a nose chamber, allowing for the concentrations of two different odorants to be controlled continuously and independently. To vary olfactory stimulation without varying perceived airflow, the carrier stream was updated dynamically to maintain a constant total flow rate of 1 L min^−1^. This carrier stream could be controlled independently to simulate variation in wind flow, though this capability was not employed here. To rapidly clear the odorants, the nose chamber covered the snout of the mouse, creating a micro-environment of volume 0.07 cm^3^ (including the snout) in which the gas volume was replaced by the 1 L min^−1^ airflow every 4 ms. The nose chamber did not touch the snout or the majority of the whiskers, and produced no obvious effect on whisking or grooming behavior (Supplementary Fig. 1)

To overcome the problems of delivery speed and consistency, we first selected two distinct odorants, methyl valerate (bubblegum smell) and α-pinene (pine smell), with vapor pressures high enough to continuously saturate the vapor phase, but low enough not to significantly deplete the liquid phase over time (11.2 and 4.9 mmHg at 25 °C, respectively). Next, we designed a rapid odorant saturation chamber in an effort to maintain steady-state saturation of the vapor phase in each odorant stream and generate perceptible but not overpowering scents at the nose chamber. For each odorant path, the air stream was bubbled through 12 mL of odorant solution (1:125 and 1:37.5 in mineral oil for methyl valerate and α-pinene, respectively) filling the bottom two thirds of a 40-mL vial that was filled nearly to the top with 3-mm glass beads. This configuration increased the interaction of air with the odorant solution first by bubbling in through the bottom two thirds of the chamber and then by passing through the increased solution surface area generated by the coated beads in the top third. We then used a photo-ionization detector (PID) to measure the response time and depletion of our olfactometer (measured at the nose chamber). When driven by 0.5-Hz sinusoids, our olfactometer delivered odorants as continuous variables with a delay of 0.148 ± 0.059 s for methyl valerate and 0.183 ± 0.070 s for α-pinene (*n* = 300 cycles: 150 low-offset and 150 high-offset) (Fig. 1b; Methods section), on the order of the sniffing cycle for mice^25^. At maximum flow rate (0.1 L min^−1^) for ~100 min, the odorant concentrations decayed with time constants of 242 and 1027 min for methyl valerate and α-pinene, respectively (Fig. 1c, Supplementary Fig. 2, Methods section). This means that over a typical behavioral session length of 30 min, the fastest-depleting odorant, methyl valerate, would be reduced in magnitude by at most 12% (if the mouse remained at the methyl valerate side of the track the whole session). Thus, we established the mechanical components capable of rapid and reliable control of odorants in VR.

### Controlling olfactory virtual landscapes

We incorporated our olfactometer into a visual VR system^5,8,9,11^ and drove the olfactometer using a modified version of existing VR software^20^. This permitted the options of synchronizing (or desynchronizing) visual and olfactory VRs, or using one or the other sensory modality alone (Fig. 1d–g).

Real-world odorant distributions can occur as concentration gradients caused by molecular diffusion, as distributed bursts of high-concentration plumes caused by turbulence, or a combination of these two resulting in concentration gradients with added noise^29,30^. Accordingly, animals have been shown to use gradient-ascent and/or sparse-searching strategies under different conditions^29–33^. To allow for the study of gradient-guided navigation, we demonstrate that our system can generate smooth spatial distributions of methyl valerate and α-pinene across a linear track (Fig. 1f). The real-world generation of odorant plumes by air turbulence is an ongoing focus of fluid dynamics research^34^, making it difficult to simulate precisely a true turbulent odorant distribution in virtual reality. However, we demonstrate that our system can generate a “noisy” odorant distribution (Fig. 1g), as well as turbulent-like plumes (Supplementary Fig. 3).

**Fig. 2:**
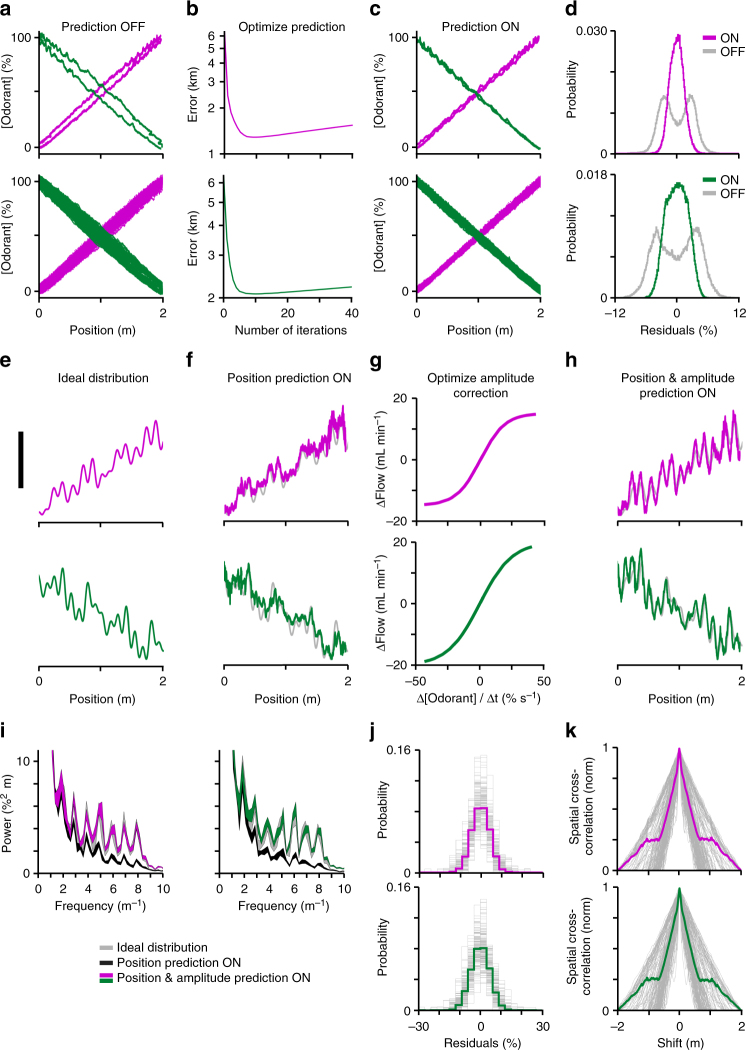
Online algorithms enhance the precision of odorant delivery. The data were acquired by replaying a recording of the same 30-min behavioral session with the PID at the nose chamber. **a**–**d** A position-predictive algorithm tightens a smooth odorant spatial distribution. **a** Odorant delivery with no position-predictive algorithm for two traversals, one toward methyl valerate and one toward α-pinene (top), and for the entire behavioral session (bottom). **b** The window for calculating instantaneous velocity for the position-predictive algorithm was optimized offline by minimizing the error between real and predicted position for each odorant. **c** Odorant delivery with the position-predictive algorithm on. **d** Residuals of the data in **a** (bottom) and **c** (bottom) to their best-fit lines. With the position-predictive algorithm off, the time lag described in **a** resulted in an odorant distribution that was skewed in the opposite direction of the run, causing a bimodal distribution of residuals (gray), one mode for each run direction. This distribution was made unimodal (color) when the position-predictive algorithm was turned on. **e**–**k** Adding an amplitude correction to the position-predictive algorithm permitted the control of a high-frequency noisy odorant distribution. **e** The ideal noisy odorant distribution for one traversal for methyl valerate (top) and α-pinene (bottom). Scale bar = 30%. **f** Odorant delivery with the position-predictive algorithm alone. **g** The amplitude correction was determined by how much extra flow was needed to achieve the concentration rate of change to generate the ideal odorant distribution (Supplementary Fig. 5). **h** Odorant delivery with the combined position-amplitude-predictive algorithm. **i** Power spectra of the ideal noisy odorant distributions (gray), and the real noisy odorant distributions with the position-only (black) and position-amplitude (color) predictive algorithms on (*n* = 205 traversals, mean ± s.e.m). Peaks occured at the ideal frequencies (integers 1–8 m^−1^) with the position-amplitude predictive algorithm on. **j** Histograms of the residuals between the real and ideal noisy odorant distributions with the position-amplitude-predictive algorithm on for each traversal (*n* = 205, gray) and for all traversals pooled (color). **k** Spatial cross-correlation plots between each real and ideal distribution (*n* = 205, gray) and averaged over all distributions (color)

Two challenges that needed to be overcome for the precise control of odorant distributions were: (1) mechanical delay of odorant delivery through the olfactometer (quantified in previous section) and (2) variable locomotion velocity of the mouse requiring rapid changes in odorant flow rates. Combined, these problems resulted in a skewed odorant spatial distribution in which the mouse received concentrations corresponding to a previous position rather than its current position (Fig. 2a). To correct for this, we developed an algorithm that delivered odorants based on a prediction of the mouse’s future position. This algorithm used the subject’s instantaneous position and velocity at each update iteration (~5 ms) of VR to predict its position at one odorant mechanical delay in the future. Instantaneous velocity was calculated by averaging the previous several Δposition/Δtime measurements within a previously determined time window determined offline by minimizing the error between real and predicted position (Fig. 2b, Methods section). Implementing the position-predictive algorithm eliminated the skew in concentration and tightened the odorant spatial distributions by factors of 4.4 and 3.7 for methyl valerate and α-pinene, respectively (Fig. 2c, d, Methods section). This position-predictive algorithm compensated for the mechanical delay of the system despite variable locomotion velocity, and thus generated precisely controlled odorant concentration gradients.

The position-predictive algorithm alone was not sufficient for precisely controlling sharp concentration changes characteristic of a noisy odorant distribution (Fig. 2e, f). This was due to two additional problems associated with the precise control of odorant distributions: (3) a variable relationship between the rate of change of MFC flow rate and the resulting odorant concentration change detected at the nose chamber one odorant mechanical delay in the future (Fig. 2g, Supplementary Fig. 5e,f), and (4) a variable odorant mechanical delay as a function of control drive frequency (Supplementary Fig. 5d). These problems, combined with problem 2 above, resulted in a noisy odorant signal that did not represent the spatial frequencies of an idealized noisy distribution when only the position-predictive algorithm was implemented (Fig. 2f). To correct for these problems, we first analyzed the frequency components of the ideal (desired) concentration distributions, calculated the mean frequency, and chose the delays (one for each odor) corresponding to this mean value. We then added an amplitude correction to our control algorithm to exaggerate the odorant stream flow rates by a magnitude that is a function of the desired concentration change (difference between current concentration and desired concentration one odorant mechanical delay in the future) (Fig. 2g). Implementing this combined position-amplitude-predictive algorithm, outlined in Supplementary Fig. 4 and 5, enhanced the frequency components of the desired concentration distribution that the position-only predictive algorithm could not achieve (Fig. 2h, i). Using this algorithm, the ideal noisy distributions were replicated with residual errors of 4.3% for methyl valerate and 4.6% for α-pinene (s.d., Fig. 2j) and spatial phase lags of 0.00 ± 0.30 cm for methyl valerate and also 0.00 ± 0.30 cm for α-pinene (Fig. 2k). We used this algorithm to control temporal frequencies up to 4 Hz (Methods section), which translates to a spatial frequency of up to 8 m^−1^ for an animal traveling at a speed of 0.5 m s^−1^. These frequencies are sufficient to simulate odorant bursts measured in a real-world behavioral chamber^33^ that occur with a duration of ~0.25 s (Supplementary Fig. 3). Thus, our final system can control noisy odorant distributions and turbulent-like plumes, in addition to smooth odorant concentration gradients.

### Olfactory-driven virtual behavior

As an initial demonstration for behavior and neural recording, we used the smooth odorant gradient track (Fig. 1f). Since olfactory-only VR has not previously existed, it is unknown whether a mouse in VR can perform purposeful odor-guided spatial behaviors, and if so, what neural systems underlie such behaviors. To determine this, we first asked whether a mouse can navigate along the virtual odorant gradients (Figs. 1f, 2c).

**Fig. 3:**
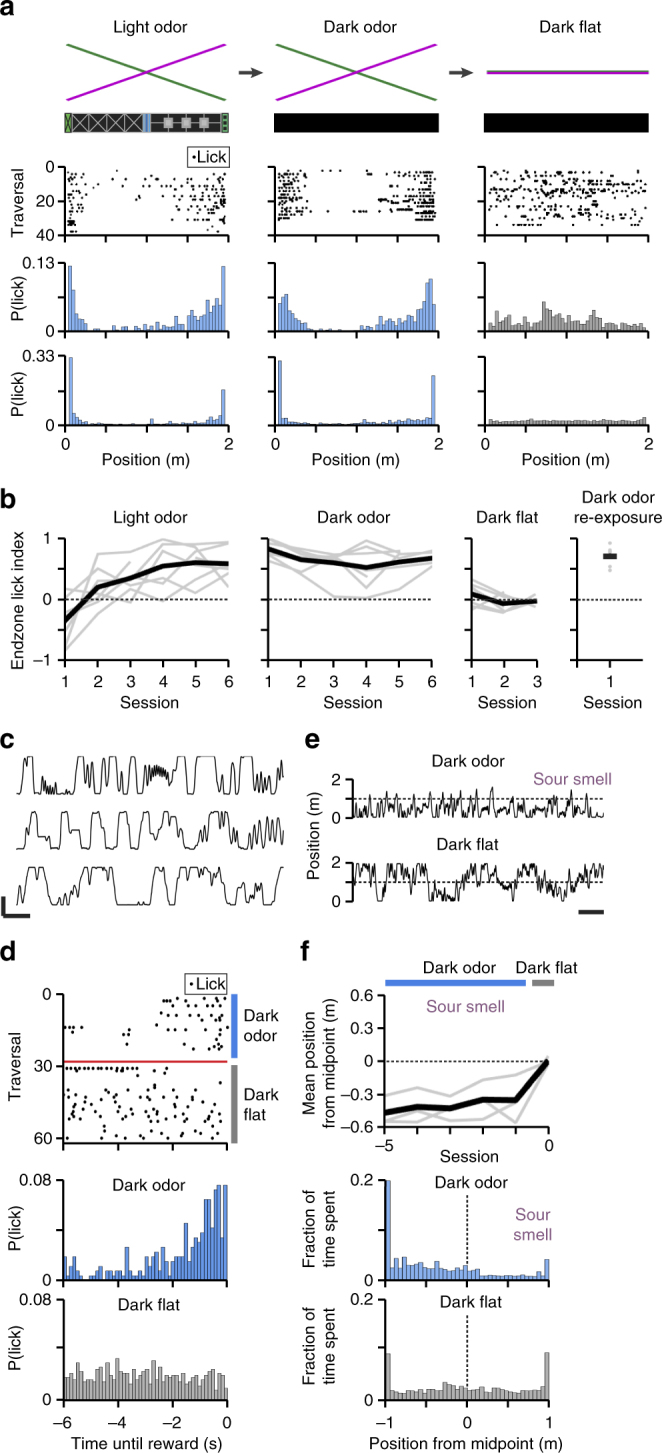
Mice can perform odor-guided behaviors in virtual space. **a** Three environmental sensory conditions (row 1). Single-session licking raster plots for the same mouse in each condition during separate sessions (row 2). Each dot represents a lick. Each row includes all of the time elapsed since the previous reward. To exclude reward consumption, licks at positions at and beyond the reward sites are not plotted. Histograms of lick position for data shown in row 2 (row 3). Histograms of lick position pooled over all 7 mice on the final session in each condition (row 4). **b** Training curves for all seven mice, measured as endzone index across days. Gaps in the curves occur where the mouse did not lick, did not achieve rewards, or had completed its training in the present condition. After sequential training in the light odor (left), dark odor (middle left), and dark flat (middle right) conditions, mice were able to perform well upon re-exposure to the dark odor condition (right). **c** Example position traces from three different mice on the final training session in the dark odor condition. Scale bar = (1 m, 1 min). **d** Temporal licking raster plot for one mouse during its first transition from the dark odor condition to the dark flat condition in the same session (top). Histograms of lick position during the transition, pooled over all seven mice (bottom). **e**,** f** In this experiment, methyl valerate was replaced with sour-smelling oxidized methyl valerate. **e** Example position trace for one mouse in its final session in the dark odor condition (top). The same mouse in the first session in the dark flat condition (bottom). Scale bar = (1 m, 1 min). The mouse avoided the sour smell in the dark odor condition. **f** Mean track positions for three mice for five sequential sessions in the dark odor condition and one session in the dark flat condition (top). Histograms of track position pooled from all three mice on sessions –1 (middle) and 0 (bottom). Peaks occur at the endzones because mice were more likely to get “stuck” there, i.e., half of the possible view angles point into the wall rather than along the track

We trained head-fixed, water-scheduled, wild type, male C57BL/6 mice to run on a spherical treadmill to traverse a virtual linear track (Fig. 1d–f) to receive a water reward at each alternating track endzone as described previously^5,8,11^. To learn the angular gain of the treadmill, mice were first trained with visual and olfactory cues (light odor condition) for ~6 days, ~30–45 min/day until they consistently performed anticipatory licking as detected by a capacitive circuit (Fig. 3a, d). This licking indicated that the mice had formed an association between track location and reward. The visual VR was then shut off for all subsequent behavioral sessions (~6 days, ~30–45 min/day, dark odor condition), forcing the mice to navigate by olfactory cues alone (Fig. 3a, c). In this condition, comparable anticipatory licking to the light odor case was observed (Fig. 3a). Interestingly, mice showed no decrease in performance on the first dark odor session (Fig. 3b), indicating that no additional training was needed to navigate in the dark odor condition. To control for the possibility that the mice in the dark odor condition were navigating using odor-independent strategies, such as path integration^35^, timing, or step-counting, the odorant gradients were then “flattened” (dark flat condition) to make the odorant concentration position-invariant (Fig. 3a). Immediately after flattening (Fig. 3d), and for the subsequent sessions in the dark flat condition (Fig. 3b), anticipatory licking was abolished (*p* = 5.8 × 10^–4^, two-sided Mann–Whitney *U*-test between endzone lick indices in the final dark odor session (0.76 ± 0.18) vs first dark flat session (0.12 ± 0.17) when instantly switched (Fig. 3d), *n* = 7 mice). This indicated that behavior in the dark odor condition was indeed odor-dependent. Thus, we established an odor-driven behavior in virtual space. Although self-motion cues alone were not sufficient to solve the dark flat task, they are necessarily present in all conditions, and are likely used in conjunction with the odors to solve the dark odor task.

Separately, we found that when using oxidized methyl valerate, a sour-smelling odor, three of four mice in a cohort consistently avoided this odor for several consecutive days in the dark odor condition (Fig. 3e, f). When transferred from the dark odor condition (mean position = 59.4 ± 14.7 cm) to the dark flat condition (mean position = 100.5 ± 3.5 cm), this place-avoidance was abolished (*p* = 9 × 10^–6^, repeated measures ANOVA, *F*(1,14) = 45.8, *n* = 3 mice) (Fig. 3e, f). This indicates that our olfactory VR system can be used to perform head-fixed assays of innate valence and place preference.

### Place cells in olfactory VR

**Fig. 4:**
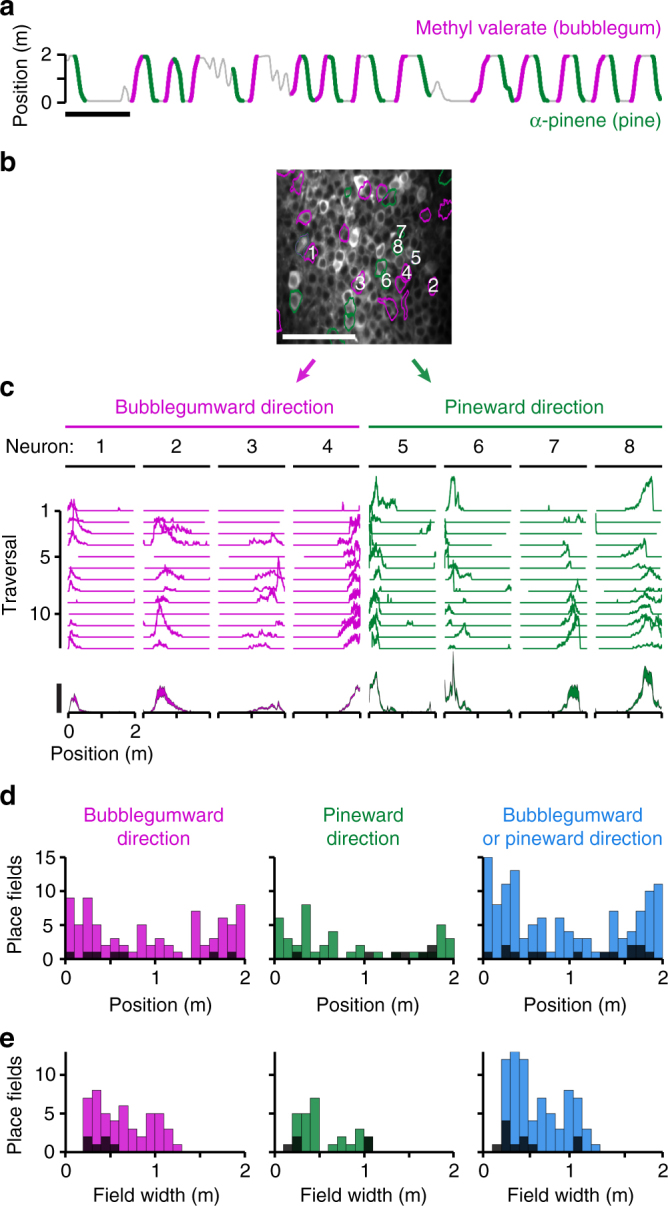
Olfactory-guided virtual navigation in darkness engages CA1 place cells. **a** Position of a mouse behaving in VR. Traversals in the bubblegumward and pineward direction are colored pink and green, respectively. Scale bar = 1 min. **b** Averaged field of view of CA1 stratum pyramidale during the behavioral session shown in **a** as measured by 2-photon calcium imaging. Each significant place cell is outlined with the color indicating the direction in which its place field occurs. Neurons with place fields in both directions are outlined in both colors. Scale bar = 100 µm. **c** Traversal-by-traversal (top, arbitrary units) and mean change in fluorescence (bottom, Δ*F*/*F*, mean ± s.e.m.) for each neuron numbered in **b** during traversals in its preferred direction as indicated in **a**. Scale bar = 100% Δ*F*/*F*. **d** Histograms of all mean place field peak locations during traversals in the bubblegumward direction (left), the pineward direction (middle), or either direction (not both directions) (right), pooled from seven mice, one session per mouse. Colored bars represent the dark odor condition and black bars represent the dark + flat condition for the same fields of view during the same behavioral sessions. **e** Histograms of all place field widths for the same data as described in **d**

To determine whether this behavior engages neural mechanisms known to be associated with real-world^36^ and visual VR^3,5,8,11,15,20^ navigation, we monitored potential “place cells” in CA1 of the hippocampus. Before training, we injected a virus to express the genetically encoded calcium indicator GCaMP6f^37^ in neurons of CA1 stratum pyramidale and implanted a chronic imaging window over CA1 as described previously^8,11^. After completing the training described above, we performed 2-photon calcium imaging of CA1 during behavior in the dark odor condition (Fig. 4a, b). From the seven mice that completed the full training, we performed one imaging session per mouse and identified 120 neurons in the dark odor condition that exhibited statistically significant place fields (Methods section) characteristic of place cells (Fig. 4c). To estimate the fraction of active neurons that were place cells, we divided the number of place cells by the number of ROIs identified by the cell-identification algorithm^38^. This fraction was 10% ± 7%, a lower value than previously reported using similar methods in visual^8,13^ and multisensory^12^ VR (Discussion).

One concern is that these place fields could be driven by an intrinsic path integration system^35^ or by firing sequences representing time or distance, as have been reported in a similar setup using a spherical treadmill with passive (computer controlled) view angle control in visual VR^19^, and also using a linear treadmill with no task-related features^39,40^. To control for this, after imaging each mouse in the dark odor condition, we instantly switched to the dark flat condition and imaged the same field of view. In this condition, the number of place cells (across the whole-imaged population) dropped drastically to 14, 88% less (Fig. 4d, e, black bars). This demonstrates that the dark odor condition spatial representation was largely odor-dependent, and not solely due to an intrinsic mechanism. Further, the firing of the place cells in the dark odor condition was associated with less variance in position than in distance run on the treadmill^40^ (Supplementary Fig. 7a).

In the dark odor condition, the spatial distribution of place fields across the track was non-uniform, as described previously for real^41^ and visual VR^8^ environments, with a greater concentration of place fields at the endzones (Fig. 4d). These place fields occurred with a width of 60 ± 30 cm (Fig. 4e), which is on the higher end of previously reported optically imaged place field widths in multisensory VR using the same indicator (~30–60 cm)^12^.

**Fig. 5:**
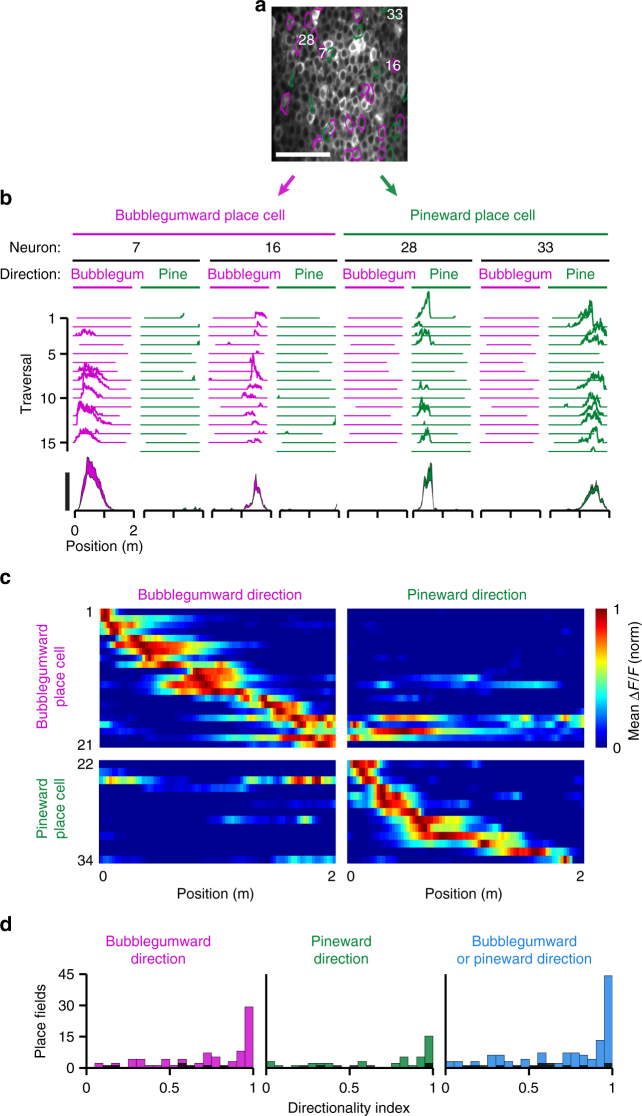
CA1 place cells during olfactory-guided virtual navigation tend to exhibit directional preferences. **a** Averaged field of view of CA1 stratum pyramidale during olfactory-guided virtual navigation. Each significant place cell is outlined with the color indicating the direction in which its place field occurred. Scale bar = 100 µm. **b** Traversal-by-traversal (top, arbitrary units) and mean change in fluorescence (bottom, Δ*F*/*F*, mean ± s.e.m). for each neuron numbered in **a** during traversals in its preferred and non-preferred direction. Scale bar = 100% Δ*F*/*F*. **c** Normalized mean Δ*F*/*F* as a function of track position for each place cell outlined in **a** during runs in its preferred and non-preferred direction. **d** Histograms of all place field directionality indices during traversals in the bubblegumward direction (left), the pineward direction (middle), or either direction (not both directions) (right), pooled from seven mice, one session per mouse. Colored bars represent the dark odor condition and black bars represent the dark flat condition for the same fields of view during the same behavioral sessions

Of the 120 place cells identified, 78 exhibited place fields during traversals of increasing methyl valerate (bubblegumward direction), 40 exhibited place fields during traversals of increasing α-pinene (pineward direction), and only 2 exhibited place fields at any locations in both directions (Fig. 4d, e). To further characterize these directional preferences, we examined each neuron’s activity in its preferred vs non-preferred direction. (Fig. 5a, b). A trajectory along one direction tended to be represented by a different population of place cells than a trajectory along the opposite direction (Fig. 5b, c). To quantify each neuron’s directional preference, we calculated a directionality index^8^ (Methods section) where 0 indicates bidirectionality and 1 indicates unidirectionality. These place fields exhibited directionality indices of 0.73 ± 0.30, indicating a strong tendency toward unidirectionality (Fig. 5d). Such directional preferences of place cells have previously been reported in real^41–43^ and visual VR^5,8^ environments. Further, the directionality indices in the dark odor condition occurred within the range of those reported for visual virtual navigation (0.83 ± 0.25)^8^. This directional preference indicates that these neurons are tuned to representations of specific trajectories^43^ rather than to lower level task features, such as concentration, reward-prediction, self-motion, distance, or timing. These results demonstrate that locomotion through odorant gradients can provide sufficient information for the CA1 network to recruit a cognitive map^44^.

### Behavior in a noisy olfactory virtual landscape

**Fig. 6:**
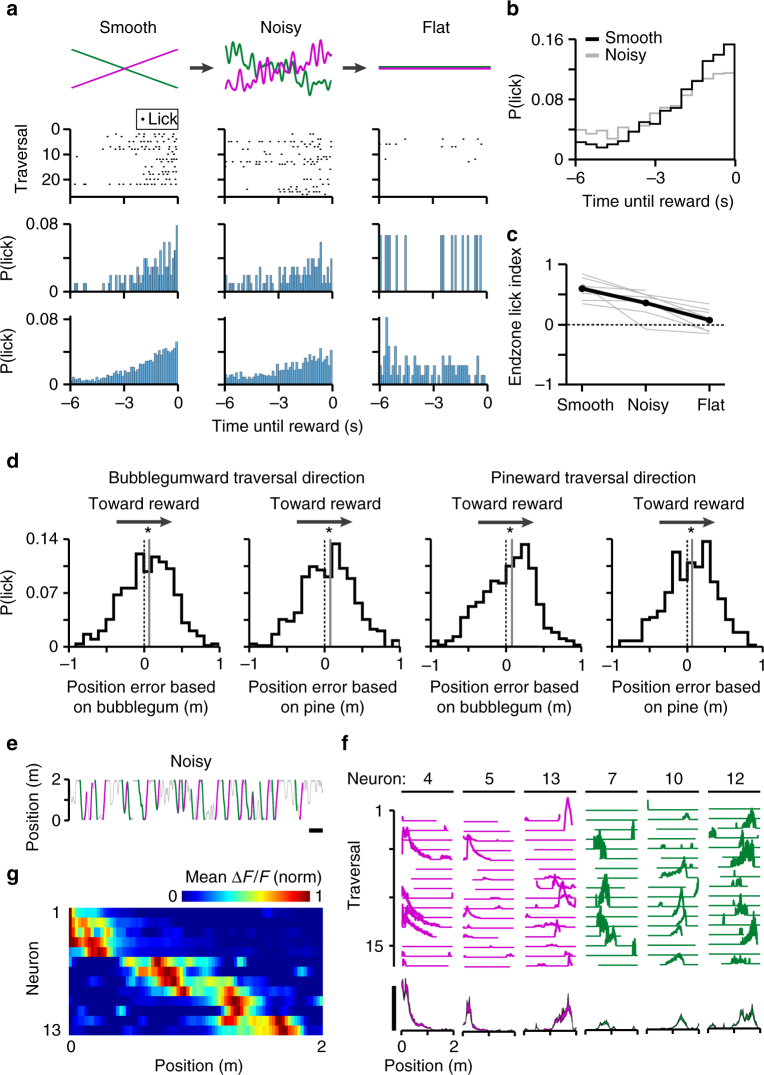
Olfactory noise affects behavior and place cells are present during noisy odor navigation. **a** Mice were switched between familiar smooth, noisy, and flat odor conditions (row 1). Example anticipatory licking raster plots (row 2) and corresponding histograms (row 3) for the same mouse in each condition. Pooled anticipatory licking histograms across all five mice in eight sessions (row 4). **b** Overlaid anticipatory licking histograms from **a** bottom. Anticipatory licking was less precise and occurred earlier in the noisy vs smooth conditions. **c** Endzone lick index (Methods section) across conditions for all sessions (gray) and averaged (black). **d** Odor-based position errors bias anticipatory licking toward the reward site. Histograms are pooled across all five mice in eight sessions. Medians (gray lines) of all distributions are significantly different form zero (dotted lines) (two-sided Wilcoxon signed-rank test, *p* = 0.0002 (first column), *p* = 0.0004 (second column), *p* = 0.0117 (third column), *p* = 0.0014 (fourth column)). **e** Position of a mouse behaving in the noisy condition. Scale bar = 1 min. **f** Traversal-by-traversal (top, arbitrary units) and mean change in fluorescence (bottom, Δ*F*/*F*, mean ± s.e.m.) for six simultaneously recorded place cells during traversals in their preferred directions as indicated in **e**. Scale bar = 100% Δ*F*/*F*. **g** Mean Δ*F*/*F* as a function of track position for place cells identified in the noisy condition. Place cells were pooled across both running directions

We next validated our system for studying behavior in a noisy olfactory virtual landscape (Fig. 1g). We trained a separate cohort of mice to navigate in the dark odor condition (smooth condition), as well as in the dark odor condition with noise added (noisy condition, Methods section). In order to add noise on top of a linear odorant spatial distribution (Fig. 1f, g), the range of the linear odorant spatial distribution was reduced to 30–70% (as opposed to 0–100% used in Figs. 3–5). Once proficient in both conditions, mice were transferred between the smooth, noisy, and flat conditions (Fig. 6a). Well-trained mice showed anticipatory licking in both the smooth and noisy conditions, but not in the flat condition, indicating that this noise level does not completely disrupt the behavior (Fig. 6a). In the noisy compared to the smooth condition, anticipatory licking was less precise: it began significantly earlier before reward delivery (Fig. 6b, smooth: −1.8 ± 1.5 s; noisy: −2.2 ± 1.7 s, *p* = 6.7 × 10^–8^, two-sided Mann–Whitney *U*-test) and the endzone lick index (Methods section) was significantly affected by odor condition (Fig. 6c, *p* = 7 × 10^–5^, repeated measures ANOVA, *F*(2,12) = 23.6, *n* = 7 sessions from five mice). These results indicate that this noise level hampered, but did not abolish, the mouse’s ability to accurately anticipate rewards. This demonstrates that our system can be used to study the effects of environmental sensory variation on mouse behavior.

We next examined the effect of the direction and magnitude of noise fluctuations on anticipatory licking (Fig. 6d, Supplementary Fig. 8). At each anticipatory lick (Methods section) in the noisy condition, we calculated position error as the difference between the mouse’s true position and its “perceived position” based on each odorant concentration level (Methods section). For example, a positive deflection of bubblegum would correspond to a position error toward the bubblegumward reward site. This analysis was performed separately for both odors and both traversal directions. In every case, position error toward the reward site corresponded to an increased chance of licking (Fig. 6d). Anticipatory licking was distributed about median position errors of +6.5 ± 33.8 cm (bubblegum odor, bubblegumward direction, *p* = 0.0002), +7.6 ± 34.3 cm (pine odor, bubblegumward direction, *p* = 0.0004), +7.9 ± 36.4 cm (bubblegum odor, pineward direction, *p* = 0.0117), and +6.4 ± 33.4 cm (pine odor, pineward direction, *p* = 0.0014) (two-sided Wilcoxon signed-rank test). This indicates that olfactory noise can systematically bias a mouse’s estimation of its position, and validates that our system can control high-frequency odor fluctuations of behavioral relevance.

As a proof of principle, we next imaged hippocampal neurons in one mouse during behavior in the noisy condition (Fig. 6e–g) and identified 13 place cells. For the control case, we identified only 1 place cell for the same field of view in the flat condition, suggesting that these 13 place cells were indeed largely odor dependent. While a thorough characterization of the noise-sensitivity of place cells will require extensive experiments exploring the parameter space of the fluctuations (e.g., amplitude, frequency, spatial vs temporal oscillations), we demonstrate in principle that behavior in a noisy olfactory virtual landscape can engage a cognitive mapping^44^ mechanism.

## Discussion

We have established a system to create and maintain precise odorant distributions across virtual space. We have also demonstrated that mice can use these odorant distributions to perform spatial behavior that engages hippocampal cognitive mapping.

Continuous odorant spatial distributions have been achieved in real-world behavioral chambers using spatially arranged airflow sources and sinks^31^ or by imaging and computationally modeling odorant point sources^45^, and these methods have been validated for studying odor-place preference and spatial navigation in small animals, such as drosophila. Similar systems have also been used in rodents to study odor-place preference^46^, odor trail-tracking^47^, and odor-guided navigation^33^. However, in such rodent systems, it is difficult to control for multisensory features of the environment, such as walls/borders and self-generated odor cues, and it is difficult to quantify odorant concentration due to real-world turbulence introduced by the airflow and by the moving rodent. One system has been designed to control concentration as a continuous variable for flies^48^, but this system has not been validated for timescales faster than ~1 s, nor has it been applied to rodents or VR. The system presented here eliminates multisensory features and precisely controls concentrations during behavior, allowing more direct relationships to be drawn between an olfactory landscape and its neural representation.

It is well-established that neurons in CA1 can respond to odors^49–57^. Perhaps due to the inability to control concentration as a continuous variable, however, research on the effect of odor on these neurons has been limited mainly to object recognition^54,57^ and contextual association^51^, with odor almost always categorized as non-spatial information^49,51,52,54,56,57^. These findings have led to valuable conceptual and circuit models of how the hippocampus processes spatial vs non-spatial information^54,58,59^. Yet, despite several ethological lines of evidence suggesting that olfactory-guided navigation was a major driving force behind the evolution of the hippocampus^60,61^, olfactory-guided navigation has scarcely been incorporated into hippocampal models. Here, by defining odorant concentrations as continuous spatial variables, we demonstrate that the CA1 place cell network is able to recruit a cognitive map even when the only spatial feature of an environment is odorant concentration (Figs. 4, 5). Whether and how this result fits into current spatial vs non-spatial models of the hippocampal system is unknown, but can be addressed in the future by combining the present method with pathway-specific circuit manipulations^59^. It is possible that the directional preferences of the place fields (Fig. 5) could be explained by the concentration gradients being perceived differently along the different trajectories, as could occur by olfactory adaptation. This potential role of olfactory adaptation in navigation can be investigated in the future by combining the present technique with recordings/manipulations of adaptation-related regions of the olfactory system^62^. It is also possible that the directional cognitive maps (Fig. 5) represent abstract trajectories through a sensory continuum^63^ rather than true trajectories through space. Nonetheless, the present results demonstrate in principle that the hippocampus can recruit a cognitive map in an odor-only environment.

Olfactory VR seems to engage fewer place cells than visual^8,13^ or multisensory VR^12^. This could possibly be explained by the fact that only a subset of CA1 pyramidal neurons (including place cells) receives direct olfactory input from lateral entorhinal cortex via the temporoammonic pathway^59^. Another study has reported fewer place cells in visual VR than in a real environment^19^, suggesting that when modalities of sensory information are subtracted, place cell number decreases. It is possible that such a mechanism could account for the low number of place cells found here in olfactory VR.

The mechanisms by which multisensory cues inform place cell representations are presently a subject of controversy^19,64^. Attempts have been made in real-world environments to determine such a mechanism by manipulating odor cues relative to visual cues^50,51,53,55^, but this approach has yet to yield a clear consensus. By a different approach, it has been shown that bats use different hippocampal maps to navigate the same environment when using vision versus echolocation^65^. Yet, this does not address how an animal’s brain represents a single environment while simultaneously using two different sensory modalities. While a full investigation of multisensory integration will now be possible in future studies, here we establish behavior in an environment defined by two and only two sensory modalities—vision and olfaction (Fig. 3a, b). This opens the doorway to performing more sophisticated multisensory experiments, such as flipping or shifting odor cues relative to visual ones. This ability to synchronize (or desynchronize) precisely defined visual and olfactory cues in isolation creates a new and straightforward way to study how multiple sensory streams can integrate to inform the neural representation of space.

How the brain produces innate attractive vs aversive responses is of great interest to cognitive neuroscience. Yet, the ability to deliver controlled stimuli to an animal while recording from its brain and evaluating its innate behavior presents a technical challenge. Using our system, the innate behavior of retreating from a noxious odor (Fig. 3e, f) can be monitored using fine stimulus control and advanced recording and stimulating techniques that are difficult to apply in state-of-the-art real-world systems^46^. Thus, the present system offers a new way to study the neural mechanisms of valence.

While this study focuses on dorsal CA1, olfactospatial processing has been shown to involve many other brain areas, including ventral CA1^52^, CA3^52,66^, postsubiculum^67^, anterior dorsal thalamus^67^, mediodorsal thalamus^68^, entorhinal cortex, amygdala^69^, and olfactory bulb^70^. Brain regions upstream of hippocampus, such as lateral entorhinal cortex^71^ and piriform cortex^72^ are of particular interest for studying how the navigation system uses odor and spatial information. The present method thus opens a vast array of possibilities for studying olfactospatial processing in these areas of the behaving animal using previously infeasible recording and stimulating methods that require head-fixation.

The ability to control sensory variability during a behavioral experiment is central to studying the neural bases of adaptive behaviors and prediction-making^73^. But in real and visual VR environments, meaningful sensory variables are difficult to quantify due to the high-dimensionality intrinsic to viewing 3D space. Thus, visual VR experiments have used sensory manipulations, such as deleting landmarks^3^, narrowing walls^4^, changing cues from “vivid” to “bland”^74^, or abruptly changing the entire visual scene^13,20^. A method to control a spatial sensory feature as a single variable has been difficult to conceptualize, even in the unisensory world of visual VR. The present system offers a clear method to control the variation of concentration as a single parameter while monitoring a spatial behavior and its neural correlates (Fig. 6). This capability provides a way forward for studying how basic features of an environment, such as sensory variability, can inform the neural representation of a behavior.

## Methods

### Olfactometer

To deliver odorants with spatial precision to the behaving mouse, pressurized air (20 PSI) was run through 1/4-inch nylon tubing to a gas purifier (Chromatography Research Supplies) and split to reach three MFCs in parallel (Fig. 1a). Two MFCs (Alicat MC-100SCCM-D, flow rate 0–0.1 L min^−1^) led to 1/32-inch Teflon tubing bubbling into a 12-mL solution of methyl valerate (Sigma-Aldrich, 1:125 methyl valerate:mineral oil) or α-pinene (Sigma-Aldrich, 1:37.5 α-pinene:mineral oil) contained in a 40-mL amber glass vial with a rubber membrane cap (Thermo Fisher). Vials were filled nearly to the top with soda-lime glass beads (diameter 3 mm, Sigma-Aldrich) to increase air bubble saturation and decrease splashing. Odorant type, concentration, and vial configuration were optimized empirically to maximize delivery speed while minimizing odorant exhaustion over long durations. Outlet tubing (Teflon, 0.002-inch) above the beads/liquid led to a custom-made Teflon mixing block where the odorant streams met a stream of blank air delivered by a third MFC (Alicat MC-15SLPM-D/10 M, flow rate 0–1 L min^−1^) through 1/32-inch tubing. This air-odorant mixture then led through 1/32-inch Teflon tubing to a custom-made Teflon nose chamber fully covering the snout of the mouse. To eliminate variation in airflow, blank air flowrate was updated dynamically to maintain a constant final flow of 1 L min^−1^. All junctions were secured by threaded Teflon fittings (NResearch) reinforced with Teflon tape, and regularly passed water-immersion leak-checks. All tubing length was minimized, and all tubing diameters were optimized to the minimum possible without compromising MFC operation or causing backflow of odorant solution into the MFCs (through capillary action). To prevent flows in any direction but toward the nose chamber, during all odorant delivery sessions, including testing and behavior, the odorant stream flow rates were never permitted to fall below 1 mL min^−1^.

Odorant measurement. Relative odorant concentration was measured using a miniature photo-ionization detector (PID, Aurora) with a flow rate of 950 mL min^−1^ placed at the nose chamber. The PID signal, all MFC command and feedback signals, and virtual position and view angle were recorded and synchronized at 1 kHz using a Digidata1440A (Molecular Devices) data acquisition system (Clampex 10.3). Before each PID measurement, relative odorant concentrations of 0 and 100% were defined as the mean of 5 s of PID signal at flows of 1 and 100 mL min^−1^, respectively. Subsequent measurements were then normalized accordingly. Since the PID cannot simultaneously distinguish two odorants, each PID test was performed separately. To control for any potential variation due to PID placement, the PID was fixed rigidly to the nose chamber for all sequences of measurements. To facilitate odorant clearance, inward- and outward-facing fans were built into the ceiling of the behavioral chamber. At the end of each day of odorant delivery, and between all PID measurements of different odorants, the odorant bottles were removed, submerged tubing was wiped clean, empty bottles were attached, and all MFCs were set to maximum flow rate for 30 min. Fresh odorants solutions in new bottles were mixed daily from stock odorants that were stored in nitrogen gas. For the odor place-avoidance experiment (Fig. 3e, f), odorants were not stored in nitrogen. This resulted in sour-smelling oxidized methyl valerate.

Speed of odorant delivery. To evaluate the speed of odorant delivery, each odorant MFC was driven by a sinusoid of 0.5 Hz of low or high offset for 150 cycles (Fig. 1b). Delay between the command and the odorant delivery was calculated as the mean peak-to-peak difference between the command signal and the PID signal. To characterize the speed of odorant delivery as a function of sinusoid frequency, we applied 10 cycles each of all combinations of sinusoids of amplitudes (10, 20, 30, 40, 50, 60, 70, 80, 90, and 99) mL min^−1^ and frequencies (0.5, 1, 1.5, 2, 2.5, 3, 3.5, 4, 4.5, and 5) Hz and calculated the delay for each sinusoid. Delay was found to be relatively amplitude-invariant at each frequency, and was thus pooled over all amplitudes and plotted as a function of frequency (Supplementary Fig. 5d). Note that this amplitude-invariant sinusoidal response is conceptually different from the amplitude-dependent square pulse response described in the section below.

Stability of odorant delivery. To evaluate the stability of odorant delivery, each odor MFC was left at its maximum flow rate of 100 mL min^−1^ for ~100 min (Fig. 1c, Supplementary Fig. 2). A problem for long-duration PID use is drift of the baseline reading. To quantify the PID signal relative to a stable baseline, we took baseline measurements (odorant stream flow of 1 mL min^−1^) for 1 min every 20 min (gaps in Fig. 1c), fitted these baseline points with a second-order polynomial, and subtracted this polynomial from the non-baseline points (Supplementary Fig. 2). After baseline correction, time constants of depletion were calculated by fitting an exponential to the non-baseline points (Supplementary Fig. 2). We speculate that methyl valerate was delivered more quickly (Fig. 1b, Supplementary Fig. 5d) but also depleted more quickly (Fig. 1c) because of its higher vapor pressure.

This system was integrated into a visual virtual reality setup, and both visual and olfactory virtual realities (Fig. 1d–g) were controlled in Matlab using the Virmen virtual reality engine^20^ with a refresh period of ~5 ms. Flow rates were updated at each iteration of VR using Matlab to send analog voltages to the MFCs via a data acquisition card (National Instruments). To create smooth odorant gradients across a 2 m linear track (Fig. 1f), we defined MFC flow rates *F* (mL min^−1^) as functions of virtual position *x* (m):

1 $$F_1 = 1 + \frac{{99}}{2}x$$

2 $$F_2 = 100 - \frac{{99}}{2}x.$$

In every testing and behavioral session, the carrier stream was set to maintain a constant flow rate of 1000 (mL min^−1^):

3 $$F_3 = 1000 - F_1 - F_2.$$

Some high-frequency noise in the odorant spatial distribution occurred at the track ends at high concentrations (Fig. 2a, c). This noise was due to two factors: increased variability of odorant delivery at high concentrations (Supplementary Fig. 6), and increased likelihood of mouse accelerations near the reward sites (Supplementary Fig. 3). It is likely that our first-order algorithm cannot completely correct for these accelerations.

### Position- and position-amplitude-predictive algorithms

To apply a position-predictive algorithm to control smooth odorant gradients (Fig. 2a–d), we first selected the time delays measured as outlined above using 0.5-Hz sinusoids. These delays were 0.148 s for methyl valerate and 0.183 s for α-pinene. These were the times it took from each command signal for each odorant to be received at the nose chamber, and therefore the times Δt in the future that the algorithms needed to predict. Since Δt was different for each odorant, two independent algorithms were implemented to control each odorant stream in parallel. Each algorithm used first-order kinematics at each iteration of VR to predict future position *x*_f_ as:

4 $$x_{\mathrm{f}} = x_0 + v_0{\mathrm{\Delta }}t,$$

where *x*_0_ is the instantaneous position and *v*_0_ is the instantaneous velocity. Since the VR physics engine itself included no acceleration component^20^, no acceleration term was included in the algorithm. *v*_0_ was calculated as the average of the preceding several Δ*x*/Δ*t* samples:

5 $$v_0 = \frac{1}{w}\mathop {\sum }\limits_{i = 1 - w}^0 \frac{{x_i - x_{i - 1}}}{{t_i - t_{i - 1}}},$$

where *w* is the smoothing window measured in number of iterations *i*. As it is not obvious how many previous iterations should be averaged to calculate instantaneous velocity, this parameter was optimized offline. To do this, we first created a standard behavior data set comprised of 10 min each of good, mediocre, and poor performance (characterized by the reward rate and distance run between rewards) chosen from our behavioral datasets at sampling rate 1 kHz (Supplementary Fig. 5a). We then replayed this testing set in VR to simulate behavior in real time, and recorded the positions and timestamps in order to achieve the same sampling rate as the VR engine (4.9 ± 0.1 ms). We then applied Equation 4 offline at each timepoint to get each predicted position *x*_f_. This was done for each value of *w* from 1 to 40. For each *w*, error was calculated as the sum of the absolute differences between all predicted positions *x*_f_ and their corresponding real future positions *x*(*t*_0_ + Δ*t*) over all *n* samples:

6 $$\rm {Error} = \mathop {\sum }\limits_{i = 1}^n |x_{\mathrm{f}} - x(t_0 + {\mathrm{\Delta }}t)|.$$

This error was plotted as a function of *w* (Fig. 2b), and the optimum window was chosen as the *w* at which the minimum error occurred. These windows were 9 iterations for methyl valerate and 10 iterations for α-pinene.

To test whether this algorithm reduces the error in the odorant spatial distribution in real time, the PID was placed at the nose chamber and the standard behavior was replayed with the position-predictive algorithm on and off for each odorant. Fresh odorant solutions were used for each replay. To again control for the slow drift intrinsic to the PID, each recording was broken into 5-min blocks and normalized to the best-fit line of PID signal vs position. These normalized blocks were then pooled. The difference between the data and the best-fit line was calculated as the sum of the absolute values of the residuals (Fig. 2d). Performance of the algorithm was visualized by plotting the differences between the data and the best-fit line, i.e., the residuals (Fig. 2d). Improvement bestowed by the algorithm was calculated as the ratio of least absolute deviations with the algorithm off vs on. To remove points during which the mouse was stationary and therefore the algorithm would be expected to have no effect, only points during which the mouse was moving faster than 0.1 m s^−1^ were included in these plots and calculations.

To simulate a noisy environment, a set of 1000 ideal noisy odorant spatial distributions was created. Each concentration distribution *C*(*x*) was defined as a line plus a sum of spatial sinusoids (Fig. 2e):

7 $$C\left( x \right) = ax + b + \mathop {\sum }\limits_{i = 1}^8 A_i{\mathrm{sin}}(2{\mathrm{\pi }}f_ix + \phi _i),$$

where line slope *a* = 20% m^−1^, line *y*-intercept *b* = 30% for methyl valerate and *a* = −20% m^−1^, *b* = 70% for α-pinene. For both odorants, each sine wave amplitude *A*_*i*_ was chosen randomly from (0:6%), each phase offset *ϕ*_*i*_ was chosen randomly from (0: 2*π*) rad, and sine wave spatial frequency *f*_*i*_ was [1, 2, 3, 4, 5, 6, 7, 8] m^−1^.

To simulate a turbulent-like environment (Supplementary Fig. 3), the track was first divided into 24 bins, for a maximum plume rate of 12 plumes m^−1^. Plumes were then randomly assigned to these spatial bins according to the probability distributions:

8 $${\mathrm{P}}(x) = 0.05 + 0.20x,$$

9 $${\mathrm{P}}\left( x \right) = 0.45 - 0.20x.$$

For methyl valerate and α-pinene respectively, such that methyl valerate plumes were more likely near *x* = 2 m and α-pinene plumes were more likely near *x* = 0 m. Plume concentration *C*(*x*) within a bin was defined as a half-cycle of a rectified spatial sine wave:

10 $$C\left( x \right) = b + \left| {A \ {\mathrm{sin}}(2{\mathrm{\pi }}fx)} \right|,$$

where offset *b* = 30%, plume amplitude *A* for each plume was chosen randomly from (0:50%), and frequency of the plume waveform *f* = 6 m^−1^ (1 cycle/2 bins). The concentration in bins without plumes was defined as the offset *C*(*x*) = *b*.

For both the noisy and turbulent-like distributions, the corresponding ideal flow distributions were calculated as:

11 $$F(x) = 1 + \frac{{99}}{{100}}C(x).$$

This assumes essentially a 1:1 relationship between flow and concentration at all positions, e.g., 100 % odorant always corresponds to the maximum flow of 100 mL min^−1^. A delay Δ*t* was then chosen from Supplementary Fig. 5d that was representative of the frequency components of the concentration distribution. We chose 97 and 98 ms for methyl valerate and α-pinene, respectively: the delays corresponding to the mean temporal frequency of 2.25 Hz. We then optimized the position-predictive time window for these delays as described above. These windows were 9 iterations for both odorants.

When implemented during VR, a new *C*(*x*) distribution was chosen each time the mouse turned around, i.e., its view angle crossed 0 or *π* rad in any direction, with the bubblegumward view angle direction defined as *π*/2 rad. The index of each chosen distribution and its corresponding timestamp were saved to a file each time a turn-around occurred. When replaying the standard behavior with the position-predictive algorithm on, this method resulted in a noisy-looking concentration distribution that did not capture the waveforms of the ideal distribution (Fig. 2f). This implied that the assumption of a 1:1 relationship between flow and concentration was not correct for fast concentration changes.

To determine the correct relationship between flow and concentration, we drove each odor MFC with a sequence of square pulses of flow amplitude Δ*F* upward from a baseline flow of 1 mL min^−1^ to (5, 10, 15…100) mL min^−1^, downward from a baseline of 100 mL min^−1^ to (95, 91, 87…1) mL min^−1^, and outward from a baseline of 50 mL min^−1^ to (1, 5, 10, 15,…100) mL min^−1^, 10 pulses each, 2 s/pulse, followed by 2 s baseline. We measured the change in PID signal Δ*C* at time Δ*t* after the onset of each square pulse (Supplementary Fig. 5e). This was done by taking the triggered average of the 10 PID signals and finding the magnitude of the PID signal at time Δ*t*. We then plotted all (Δ*F*, Δ*C/*Δ*t*) pairs on the same graph for all directions and amplitudes. These plots include both the upstroke and downstroke of each square pulse. The regions of this plot with Δ*F* between ~ −25 and +25 mL min^−1^  showed a linear relationship. Thus, the nonlinear regions were discarded and the linear region for each odor was fitted with a line with *y*-intercept zero (Supplementary Fig. 5f) of the form:

12 $${\mathrm{\Delta }}F = m\frac{{{\mathrm{\Delta }}C}}{{{\mathrm{\Delta }}t}}.$$

The slopes *m* of these lines were 0.512 and 0.567 (mL min^−1^)/(% s^−1^) for methyl valerate and α-pinene, respectively. This is the change in flow needed to achieve a 1% concentration change in delay time Δ*t*. These linear fits were suitable for producing turbulent-like distributions (Supplementary Fig. 3), but resulted in overshoots of the ideal concentrations for the noisy distributions (data not shown). To counteract this overshooting effect, these linear fits were replaced with logistic (sigmoidal) fits with *y*-intercept zero (Fig. 2g) of the form:

13 $$\Delta F = \frac{L}{{1 + e^{ - k(\Delta C/\Delta t)}}},$$

where (*L* = 15, *k* = 0.106) for methyl valerate and (*L* = 20, *k* = 0.080) for α-pinene.

Flow correction was then implemented during VR (first by replaying the standard behavior for testing and calibration, and subsequently online during mouse behavior). At each iteration, the predicted position *x*_f_ was calculated using Equation 4 (Supplementary Fig. 5g). The corresponding future ideal concentration C(*x*_f_) from the current ideal noisy distribution was looked up (Supplementary Fig. 5h) from the previously defined known distribution (Equation 7). The current ideal concentration *C*(*x*_i_) was subtracted from *C*(*x*_f_) to get change in concentration Δ*C*. The corrected flow rate Δ*F’* needed to achieve the future ideal concentration was then calculated using the ideal flow rate and the flow correction (Supplementary Fig. 5i) as:

14 $$\Delta F {\prime} = F\left( {x_0} \right) + \Delta F.$$

This resulted in a flow rate that preemptively “overshot” that of the ideal flow distribution (Supplementary Fig. 5j) and restored the frequency components of the ideal concentration distribution (Fig. 2h, i). To quantify these differences in noise frequencies controlled by the position-predictive vs the position-amplitude-predictive algorithms, we first selected all periods of movement of at least 0.5 m, far enough to sample at least 1/4 of the noisy distribution. For each such period, we binned (averaged) the real and ideal odorant distributions into spatial bins of 1 cm to achieve the same spatial sampling rate, then calculated the spatial power spectral density. Over all of these runs through noisy concentration distributions, we calculated the average power as a function of spatial frequency for each algorithm case (Fig. 2i). To eliminate unwanted variability, the behavior and the sequence of noisy distribution choices were set to be identical for each algorithm case.

To quantify the performance of this position-amplitude-predictive algorithm for producing noisy odorant spatial distributions, residuals were calculated between each real and ideal distribution for each traversal (Fig. 2j, gray) and for the entire replayed behavioral session (Fig. 2j, color). As the DC offset of the real odorant signal was subject to PID drift and odorant depletion (Supplementary Fig. 2), real and ideal traces on each traversal were aligned by varying the real DC offset until the sum of absolute differences between the real and ideal curves was minimized. Residuals were then calculated. To quantify relative spatial phase between the real and ideal odor curves, the cross-correlation between the real and ideal curve for each traversal was then calculated (Fig. 2k, gray). For presentation, these cross-correlation curves were normalized and averaged (Fig. 2k, color). Spatial phase lag for each traversal was defined as the peak of the cross-correlation.

### Surgery

All experiments were approved by the Northwestern University Animal Care and Use Committee. Male C57BL/6 mice of postnatal age ~70 days were anaesthetized with 1–2% isoflurane, and a craniotomy of diameter ~1 mm was made over the right hippocampus at 1.8 mm lateral, 2.4 mm caudal of Bregma. To express GCaMP6f^37^ in CA1 stratum pyramidale as described previously^11^, ~60 nL of titer 8.95 × 10^12^ GC/mL AAV1.Syn.GCaMP6f.WPRE.SV40 was injected through a beveled glass micropipette at a depth of 1.25 mm below the surface of the dura. Mice were then water-restricted to 0.8–1.0 mL/day for the rest of the experiment. After 3–7 days, a stainless-steel cannula with a glass coverslip fixed to one side was implanted over the hippocampus and a titanium headplate and light-blocking ring was cemented to the skull as described previously^8^.

### Behavior

Mice were head-fixed by the headplate on a spherical treadmill^5,8,11,75^ facing a 5-panel monitor setup^9^. An optical computer mouse (Logitech) was mounted at the equator of the front of the treadmill to record its pitch and yaw velocities as described previously^5,8,11^. Optical mouse signals were read at 1 kHz using Labview 10.0 on a separate computer, then sent to the VR engine computer via a data acquisition card (National Instruments) and read in Matlab at each iteration of VR. Gains of the optical mouse were set in the VR engine to create a track length of 2 m in the pitch rotation direction and an angular component of 7.5 treadmill revolutions in the yaw rotation direction per VR revolution. To smooth linear and angular movements in VR, the optical mouse readings were averaged over the preceding 50 ms. Averaged optical mouse readings were multiplied by their respective gains to generate virtual translations and rotations as described previously^20^. As the treadmill’s roll velocity was not recorded, virtual position and distance run on the treadmill (Supplementary Fig. 7a) were calculated using only the pitch and yaw components. MFCs were mounted on the optical table beside the treadmill so as not to obscure the mouse’s vision (Fig. 1d). The tubing, mixing chamber, and nose chamber were fixed to a steel rod. The cylindrical nose chamber of inner diameter 5.0 mm and depth 5.2 mm was positioned to enclose the mouse’s snout as deeply as possible without touching it. A water spout was placed within reach of the mouse’s tongue, and a capacitive circuit was used to register licking. All visual cues, MFC flow rates, and water rewards were controlled using a Matlab-based VR engine^20^.While a linear treadmill^3,9,13,40^ or spherical treadmill with passive (computer controlled) view angle control^19^ would have likely facilitated behavioral training, we intentionally chose the more difficult spherical treadmill with active (animal controlled) view angle control in an attempt to create a task that could not be solved by path integration alone. We chose this design for the following purposes: (1) to establish an odor-guided navigation behavior that is not simply distance-estimation, and (2) to acquire a data set of “place cells” that are indeed sensory-driven and are not only driven by an intrinsic distance^19,40^ or time^39^ metric.

Training. Repeated measures designs were used such that each animal served as its own control across conditions, thus group randomization was not necessary. Sample sizes were chosen to measure experimental parameters reliably while remaining in compliance with ethical guidelines to minimize the number of animals used, which was similar to sample sizes described previously for 2-photon calcium imaging in VR^8,9,11–13^. Experiments did not involve blinding. At least 3 days after cannula implantation, mice were placed nearly daily into the behavioral apparatus for 30–45 min in the light odor condition (Fig. 3a, b) in which rewards of 4 µL could be achieved by moving to each alternating track endzone as described previously for visual VR^5,8,11^. Smooth odorant gradients (Equations 1–2, Figs. 1f, 2c) were applied using the position-predictive algorithm (amplitude correction was not required for the smooth gradient VR). After 6–11 sessions in the light odor condition, mice exhibiting anticipatory licking were transferred to the dark odor condition. For 6 of 7 mice, this transfer occurred by shutting the lights off in the middle of the final light odor session, and for one mouse, this transfer occurred the next day. In both cases, there was no impairment in anticipatory licking on the first dark odor session (Fig. 3b). After 4–8 sessions in the dark odor condition, mice were instantly switched to the dark flat condition (Fig. 3e, f), and returned to the dark flat condition for the subsequent 1–2 sessions. This timepoint marked the completion of the behavioral training. Mice that failed to perform anticipatory licking for several consecutive days in the light odor or dark odor condition were removed from the study. Of the 18 mice we attempted to train, 13 completed the training. Of these 13 mice during the behaving imaging session (see below), 4 showed poor behavior (low track traversal rates), 2 showed poor imaging quality, and 7 showed acceptable behavior and imaging quality. Data from these 7 mice are presented in (Figs. 3–5), with the exception of (Fig. 3e, f). The valence assay (Fig. 3e, f) was performed on a separate cohort of four mice, three of which showed a consistent side preference and are therefore included in (Fig. 3d). For this valence experiment only, the optical mouse was mounted beneath the treadmill and set to an angular gain of three treadmill revolutions in the roll direction per VR revolution.

In the dark odor condition, the only sources of angular information are the rate of change of odorant concentration and the motor patterns learned during the light odor condition when visual angular cues were present. We were unsuccessful at training naive mice to perform in the dark odor condition (i.e., without prior experience in the light odor condition; data not shown). This suggests that good behavior in the dark odor condition was driven by the available odor information combined with an intrinsic path integrator that was “calibrated” in the light odor condition.

Noise experiment. For the experiment shown in Fig. 6, mice were trained as described above for 5–10 sessions in the light odor condition with the odor gradient ranges reduced to 30–70%. Once proficient in the light odor condition, mice were transferred to the dark odor (smooth) condition and trained for an additional 3–10 sessions. Once proficient in the smooth condition, mice were transferred to the dark odor (noisy) condition for 3–5 sessions in which noisy odorant spatial distributions were presented as governed by Equation 7 and controlled by the position-amplitude-predictive algorithm with a sigmoidal correction (Equation 13, Fig. 2g, h). During the last ~5–10 min of the final ~2–3 noisy sessions, mice were transferred to the dark flat condition. Once proficient in the noisy condition, the training was complete. On subsequent sessions, mice were transferred between the smooth, noisy, and flat conditions on the same day (Fig. 6a). Mice that failed to consistently perform anticipatory licking for several consecutive days in any (excluding flat) condition were removed from the study. Of the 19 mice trained, 5 achieved proficiency. Of these mice, good behavior was achieved for eight sessions by five mice (Fig. 6a–d). For one session, the flat condition was not included (Fig. 6c), so this session was removed from the ANOVA calculation. For one of these mice, CA1 imaging was performed as described above (Fig. 6e–g).

Odor-based position error. To quantify the position error associated with a given odor noise level (Fig. 6d Supplementary Fig. 8), we first calculated “perceived position” using the noisy odorant concentration C by applying the same line equation that was used to define the smooth odorant spatial distribution:

15 $$x_{{\mathrm{perceived}}}\left( C \right) = - 1.5 + \frac{2}{{40}}C.$$

For methyl valerate and

16 $$x_{{\mathrm{perceived}}}\left( C \right) = 3.5 - \frac{2}{{40}}C.$$

For α-pinene. This is the position that a mouse would perceive itself to occupy assuming that it uses a direct mapping of smooth odorant concentration to position. For example, if a mouse in the middle of the track experienced high-amplitude bubblegum noise, it would perceive itself to be on the bubblegumward side of the track. Since noise for the two odorants was not synchronized, the two odorants could produce different perceived positions at the same time. The error between perceived and true positions were calculated to obtain odor-based position error for each odor:

17 $$x_{{\mathrm{error}}}\left( C \right) = x_{{\mathrm{perceived}}} - x_{{\mathrm{true}}}.$$

This is the error in the mouse’s estimation of its position based on the odor. To calculate the effect of odor-based position error on anticipatory licking, we first defined an anticipatory lick as any lick that occurred less than 6 s before a reward and <1 m away from a reward. The two reward sites were analyzed separately. For each anticipatory lick before each reward site, we calculated the position error based on each odor. For display purposes (Fig. 6d, Supplementary Fig. 8), positive position error was defined as toward the reward.

Endzone lick index. To quantify anticipatory licking as a single value, the track was divided into sections: the middle half and the endzone quarters. These sections did not include the reward sites and beyond. The number of licks in each section was counted as *L*_m_ and *L*_e_. Endzone lick index *E* was defined as: *E* = (*L*_e_−*L*_m_)/(*L*_e_ + *L*_m_), where −1 indicates exclusively middle licking, +1 indicates exclusively endzone licking, and 0 indicates equal middle and endzone licking.

### 2-photon imaging

On the session after completing the behavioral training, mice were placed back into the dark odor condition. Mice that began to perform long track traversals with anticipatory licking were immediately subjected to 2-photon calcium imaging of CA1 stratum pyramidale. This was done using a customized Moveable Objective Microscope (Sutter Instruments) described previously^9^, except here a 40×/0.8 NA water immersion objective (LUMPlanFL N×40/0.8 W, Olympus) and ScanImage 5 was used. Laser average power at the sample (after the objective) was 30–50 mW. Time series movies of 16,000 frames, 512 × 256 pixels, 0.0675 ms/line were acquired at 57.87 Hz. A Digidata1440A (Molecular Devices) data acquisition system (Clampex 10.3) was used to record and synchronize VR position, view angle, the three MFC flow rates, licking, reward delivery, and two-photon image frame timing at 1 kHz. Mice that achieved ≳10 rewards in the dark odor condition were instantly switched to the dark flat condition, and the same field of view was imaged again after at least ~2 min in the new condition. Imaging sessions during which the mouse achieved ≳10 rewards in both conditions were analyzed for place fields. Mice that did not achieve sufficient rewards in both conditions were subjected to the same imaging protocol on subsequent sessions.

### Imaging analysis

All data analysis was performed using custom scripts written in Matlab (MathWorks). All data in the text and figures are presented as mean ± s.d. except the spectral densities presented in Fig. 2i and the mean place fields presented in Figs. 4c, 5b, and 6f where error represents s.e.m.

Movie processing. In this and the following sections of the Methods, F refers to fluorescence and not flow rate (as above). Calcium imaging movies were corrected for motion artifact using a whole-frame cross-correlation algorithm described previously^8,9,11^. ROIs were defined using a PCA/ICA algorithm described previously^38^ (mu = 0.6, 150 principal components, 150 independent components, s.d. threshold = 2.5, s.d. smoothing width = 1, 100 pixels < area of ROI < 1200 pixels; see Mukamel et al.^38^ for parameter definitions). This algorithm was applied separately for each left/right half of each movie. ROIs of opposite halves sharing a middle border were stitched together as a single ROI and their time series were averaged under the following circumstances: (1) Their temporal Pearson’s correlation was >0.7, and (2) the number of border pixels shared by both cells was >50% of the average of the number of border pixels occupied by each cell separately.

Calcium transient identification. For each ROI, a Δ*F*/*F* versus time trace was generated as described previously^8,9,11^. Briefly, slow changes in the fluorescence traces were removed by examining the distribution of fluorescence in a 20.7 s interval around each sample in the trace and normalized by the 8th percentile value. These baseline-corrected traces were then subjected to the analysis of the ratio of positive- to negative-deflecting transients of various amplitudes and durations described previously^75^. We used this analysis to identify significant transients with < 0.1% false positive error rates. The behavioral data were then binned (averaged) to the imaging frame rate of 57.87 Hz and synchronized with the corresponding significant transient traces. These significant transients were used for all subsequent analysis. Example significant transients are shown in (Figs. 4c, 5b, and 6f).

Place field identification. Each traversal direction (bubblegumward or pineward) was treated separately for the identification of place fields. We used a slightly modified version of an existing procedure^8^ to conservatively identify place fields. To exclude periods when the mouse was not actively engaged in the task, we selected for periods during which the mouse was traversing the track (Figs. 4a, 6e) as follows. To allow for slight stoppages and turn-arounds, position was smoothed with a mean filter of 5.9 s. Traversals were defined as sequences of the (non-smoothed) position during which the mouse moved with a (smoothed) speed of at least 1 cm s^−1^ for a (smoothed) distance of at least 130 cm. Only points of forward progress were included in each traversal. Δ*F*/*F* of each neuron was then plotted for each traversal of each direction (Figs. 4c, 5b, 6f). These traversal-by-traversal traces were then averaged over 80 track bins to calculate the mean Δ*F*/*F* (Figs. 4c, 5b, 6f). The mean Δ*F*/*F* was then smoothed (averaged) over a window of 3 bins. The baseline of each mean Δ*F*/*F* was calculated as the mean of the 20 bins of lowest amplitude. Potential place fields were identified as contiguous regions of the mean Δ*F*/*F* with amplitude >25% of the difference between the peak mean Δ*F*/*F* and the baseline Δ*F*/*F*. Only place fields that satisfied the following criteria were included: (1) a width of at least 15 cm and at most 125 cm, (2) a maximum mean Δ*F*/*F* of at least 0.1, (3) a mean in-field amplitude of at least 8× the mean out-of-field amplitude, (4) transients occurring during more than 30% of the traversals through the field, (5) at least 5 total traversals through the field. In the event that one neuron exhibited multiple potential place fields in the same direction, only the one with the greatest number of in-field transients per traversal was considered. In the case of a tie, only the one with the largest amplitude was considered. Potential place fields were then tested for significance. This was done by breaking the Δ*F*/*F* trace during the traversals into at least 100 segments (though leaving each individual transient intact), shuffling these segments, calculating the shuffled mean Δ*F*/*F*, and subjecting it to the same potential place field criteria as above. The bootstrapped *p*-value was calculated as the ratio of shuffled potential place fields per 1000 shuffles. Place fields with a *p*-value <0.05 were called significant place fields.

Place field properties. Each significant place field’s position (Fig. 4d, Supplementary Fig. 7b) was defined as the track position at which its peak mean Δ*F*/*F* occurred. Place field width (Fig. 4e) was calculated as the distance between the place field boundaries defined above. Any place field with a boundary at either track end was excluded from this calculation. To calculate directionality index^8^ for each significant place field (Fig. 5d), the mean Δ*F*/*F* *M* in the place field was calculated in the bubblegumward (*M*_b_) and pineward (*M*_p_) directions. Directionality index was was defined as |*M*_b_−*M*_p_|/(*M*_b_ + *M*_p_), where 0 indicates identical activity in both directions, and 1 indicates activity in only one direction.

### Data availability

The data and code that support the findings of this study are available from the corresponding author upon reasonable request.

## Electronic supplementary material

Supplementary Information
