# Supplementary material for: An olfactory virtual reality system for mice
Source: Nat Commun. 2018 Feb 26;9:839. doi: 10.1038/s41467-018-03262-4 (PMC5827522; doi:10.1038/s41467-018-03262-4)
Supplement: Supplementary file 1 — Supplementary Information [file 41467_2018_3262_MOESM1_ESM.pdf]

An olfactory virtual reality system for mice

Radvansky & Dombeck

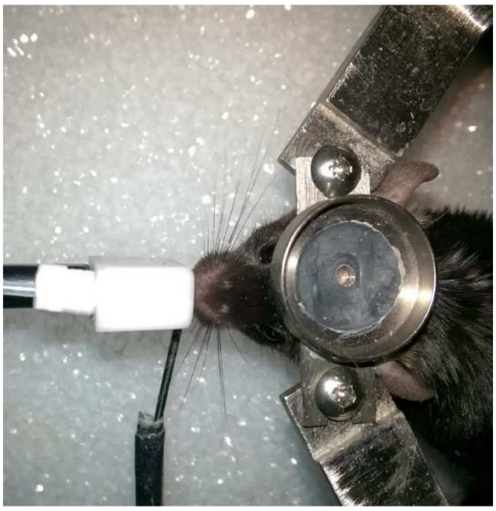

Supplementary Fig. 1: Photograph of a mouse in the olfactory VR apparatus. The mouse was head-fixed above a spherical treadmill. The head bar was ~1 inch above the treadmill, leaving enough space for the mouse to move its limbs freely. The water reward spout was positioned under the mouth. The nose chamber (white) covered the nose and snout. The nose chamber did not touch the majority of the whiskers and produced no obvious effect on whisking/grooming behavior.

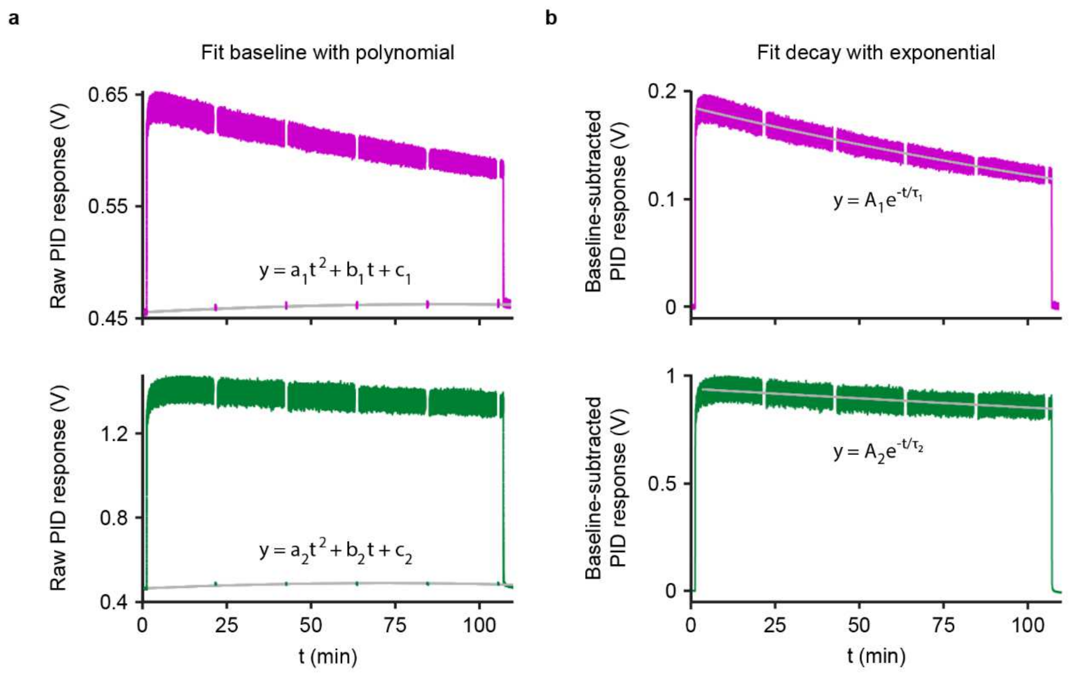

Supplementary Fig. 2: Quantifying long-term odorant delivery. These are the raw data used to create Fig. 1c. The PID was placed at the nose cone with maximum odorant flow rate of 100 mL min^{-1}, interleaved with baseline measurements of 1 mL min^{-1} for 1 minute every 20 minutes. **a,** Correction for PID drift for methyl valerate (top) and α-pinene (bottom). The baseline points were fitted with a second-order polynomial (gray). Note that in order to isolate only stable baseline values, points of transition between baseline and maximum flow were removed, and only the final 10 s of each baseline measurement was considered. The resulting constants were: $a_1 = -8.9 \times 10^{-7}$ V*min^{-2}, $b_1 = 1.6 \times 10^{-4}$ V*min^{-1}, $c_1 = 0.45$ V; $a_2 = -4.9 \times 10^{-6}$ V*min^{-2}, $b_2 = 7.0 \times 10^{-4}$ V*min^{-1}, $c_2 = 0.46$ V. **b,** Quantification of odorant decay time constant. The baseline polynomial curve in **a** was subtracted from the odorant curve to get the baseline-subtracted PID response. The remaining non-baseline points were then fitted with an exponential curve (gray) to determine the decay time constants. The resulting constants were: $A_1 = 0.19$ V, $\tau_1 = 242$ min, $A_2 = 0.94$ V, $\tau_2 = 1027$ min.

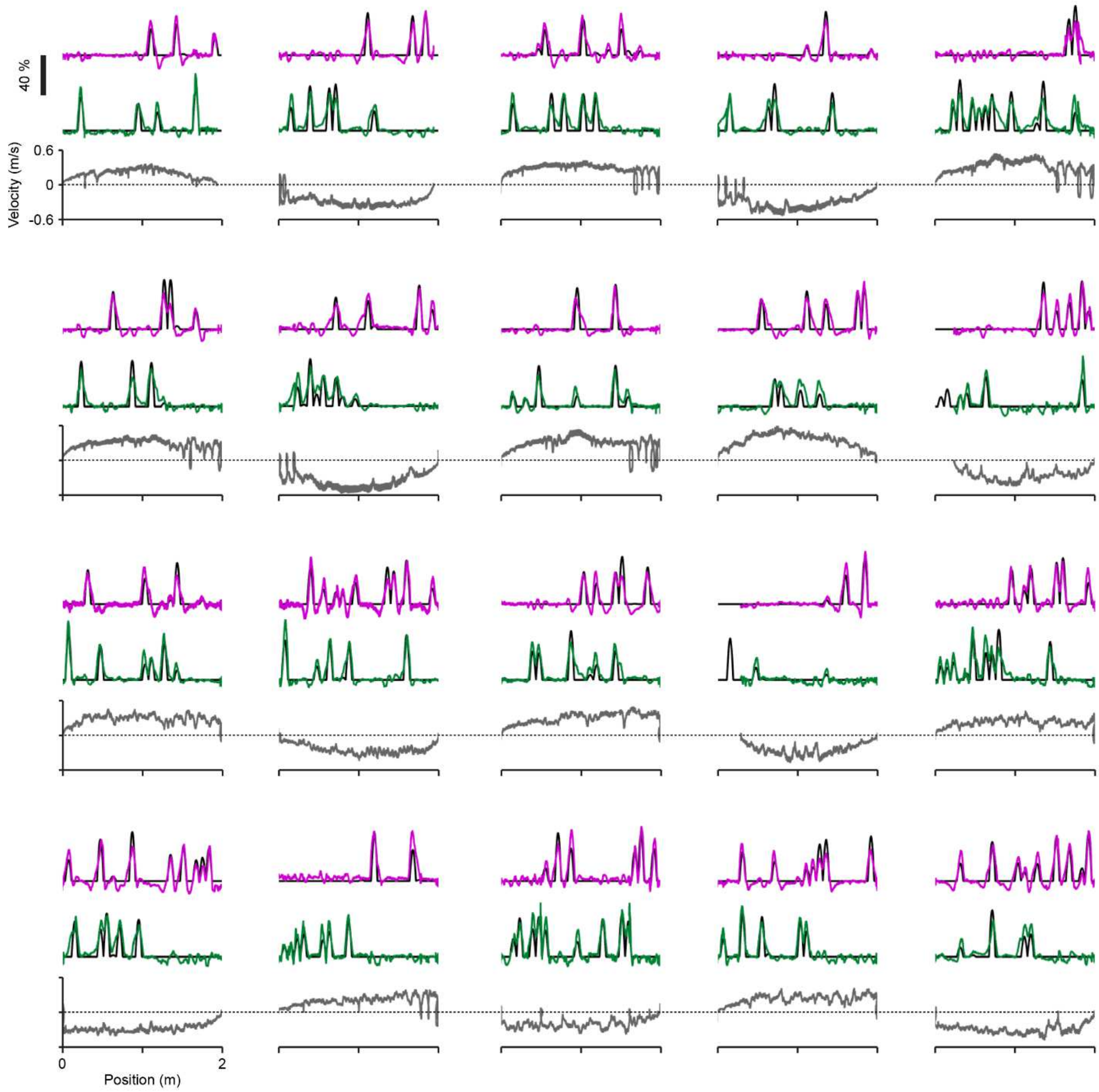

Supplementary Fig. 3: Odorant plume delivery during virtual track traversals. Each panel shows data from a different replayed traversal of the testing set (Supplementary Fig. 5a) with the PID placed at the nose chamber. The upper two traces show odorant spatial distributions for idealized plumes (black) overlaid with PID measurements for methyl valerate (pink, top) and and α-pinene (green, middle). The bottom trace shows the mouse's velocity as a function of track position, smoothed with a window of 50 ms (gray, bottom).

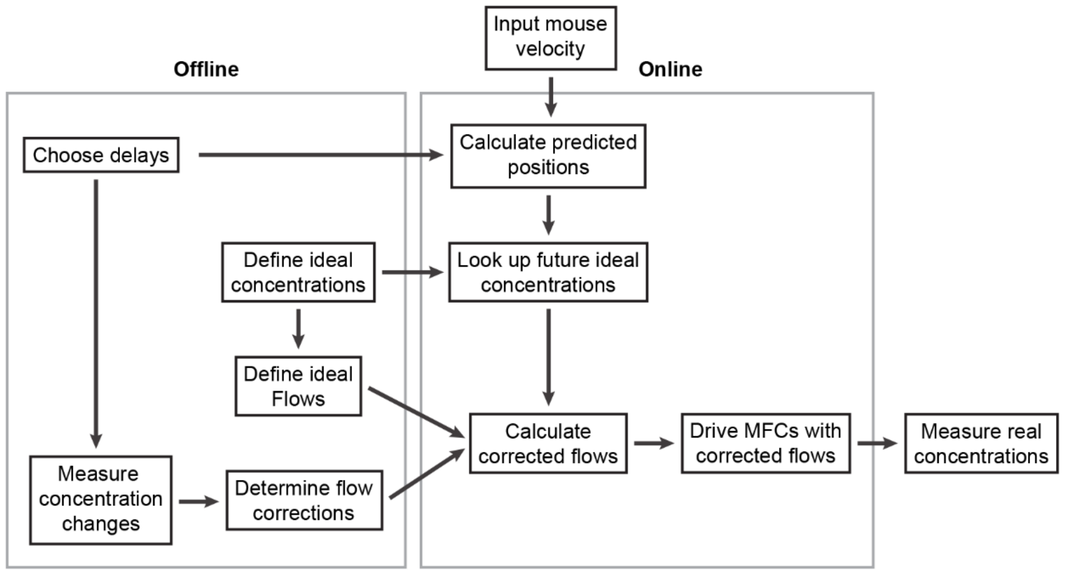

Supplementary Fig. 4: Block flow diagram of the position-amplitude-predictive algorithm. Each block is described in detail in (Supplementary Fig. 5). Offline, delays and flow corrections were measured using the PID; ideal concentrations and flows were defined by the user. These values were then fed into the online algorithm that updated at each iteration of VR during mouse behavior. Online, mouse velocity was used to calculate predicted position at one delay in the future. The ideal concentrations at this predicted position were looked-up from the previously-defined ideal concentration distributions. For smooth concentration gradients, no further correction was necessary (position-predictive algorithm, Fig. 2a-d). For sharply-changing concentrations at fast running speeds, the uncorrected ideal flows were insufficient for achieving the future ideal concentrations (Fig. 2f). Therefore, the future ideal concentrations were achieved by boosting the ideal flows by the previously-determined flow corrections. These corrected flow rates were sent as voltage commands to the MFCs. The resulting odorant concentrations could be measured at the nose chamber using the PID.

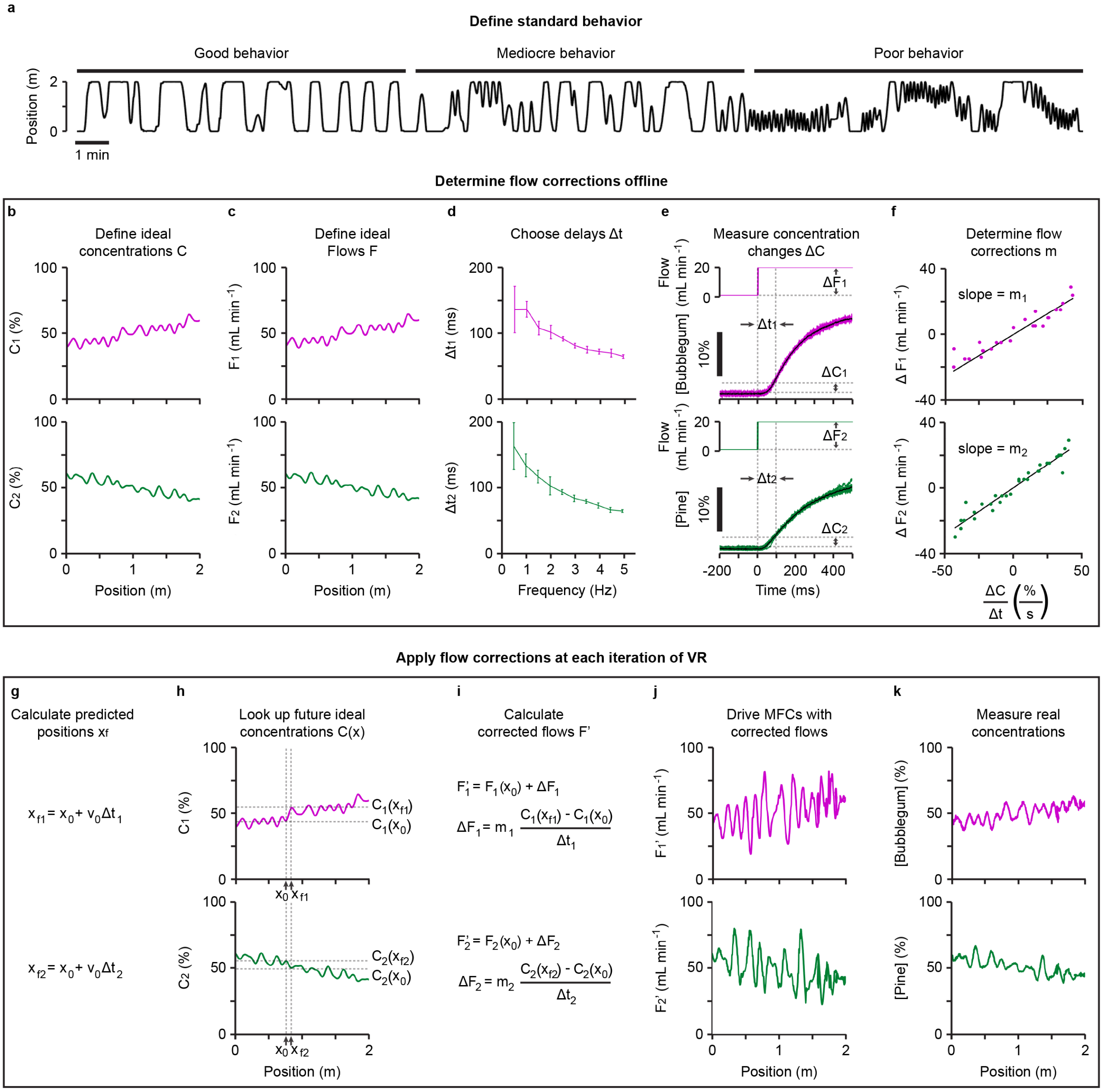

Supplementary Fig. 5: Step-by-step outline of the position-amplitude-predictive algorithm for methyl valerate and α-pinene, simultaneously. a, Define a standard testing set comprised of 10 min each of good, mediocre, and poor mouse behavior. To control for behavioral variability when comparing algorithm performance, this standard behavior is replayed in real time in VR under each algorithm case. **b,** Define the ideal concentration distributions to be created. **c,** Define the ideal flows as 10*C_i. These are the flows that would produce the ideal concentration distributions in the absence of any mechanical constraints. **d,** Choose the delays that best

represent the frequency components of the ideal concentration distributions. These plots were made by applying 10 cycles each of sinusoids of frequencies [0.5, 1.0, 1.5, 2.0, 2.5, 3.0, 3.5, 4.0, 4.5, 5.0] Hz and amplitudes [10, 20, 30, 40, 50, 60, 70, 80, 90, 99] mL min^{-1}, and calculating the mean peak-to-peak delay between the MFC command and the PID signal. Mean delays were relatively amplitude-invariant, and were thus pooled over all amplitudes. **e,** Measure the change in concentration that occurs Δt after a change in flow of a given amplitude. Concentration plots show 10 triggered signals (color) overlayed with their averages (black). **f,** From the data in **e**, plot change in flow as a function of the slope of the concentration change. These plots include flow steps upward from 1 mL min^{-1}, downward from 100 mL min^{-1}, and outward from 50 mL min^{-1}. Beyond the ranges shown, the plots became non-linear, and such points were not used for this algorithm (and rarely occurred during behavior sessions). The slope m of the best-fit line with y-intercept forced to be 0 is the amount of flow change needed to produce a given concentration change by time Δt. For some applications, this linear fit results in an overshoot of the ideal concentrations. This overshoot can be counteracted by instead fitting the points with a logistic function (Fig. 2g), thus including a saturation component to the change in flow rate. **g,** Use the mouse's initial position and velocity to predict its future position at each Δt in the future. **h,** Determine the concentration at each future position by looking up the values from the ideal concentration distributions shown in **b. i,** Use the m-values from **f** to calculate the flows needed to reach the ideal concentrations at Δt in the future. **j,** Drive the MFCs with the flow rates determined in **i**. These plots are the command signals to the MFCs for one traversal of replayed behavior through the ideal concentration distributions shown in **b**. **k,** Measure the resulting concentrations at the nose chamber. These are the PID signals during the traversal replayed in **j**.

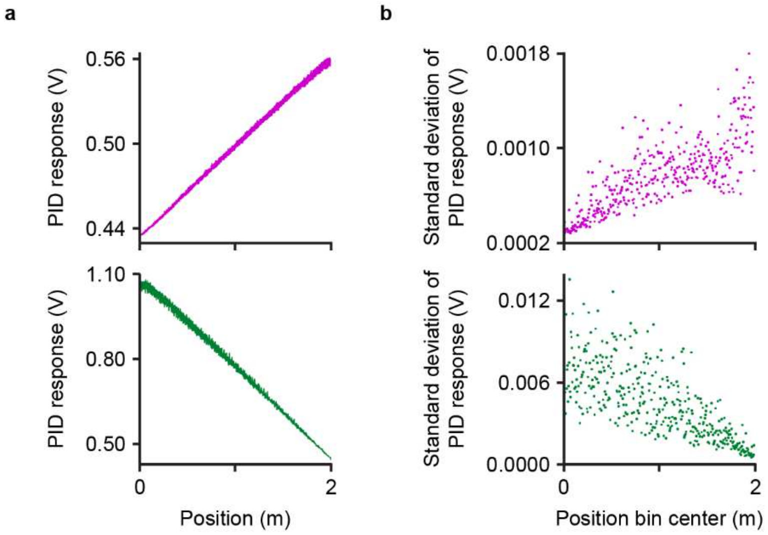

Supplementary Fig. 6: PID-measured odorant variability increases at high concentrations. **a,** PID response to a single simulated track traversal at a constant slow velocity of 0.02 m s^{-1} for methyl valerate (top) and α-pinene (bottom). The variability of each odorant distribution increased at high concentrations. **b,** Standard deviation of the PID responses in **a** as a function of track position (bin width = 0.5 cm). The PID response became more variable as the position approached the high-concentration track end for each odorant.

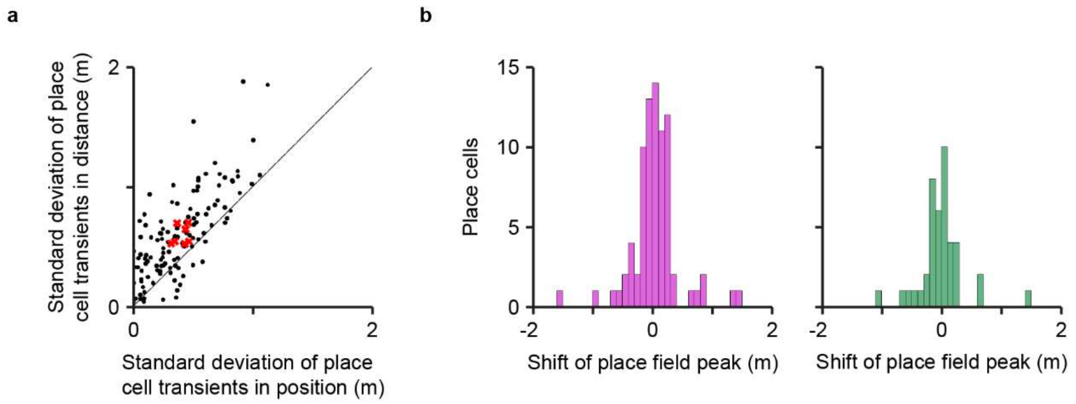

Supplementary Fig. 7: Place cell firing variability and stability. For the place cells from 7 mice reported in Figs. 4 and 5. **a,** The locations of place cell calcium transients tended to be less variable in track position than in total distance run on the treadmill. Each black dot represents one place cell. Each red x represents the mean for all place cells from one mouse. Each distance = 0 point was defined at the start of a good traversal in the place cell's preferred direction (Fig. 4a, 6e). Standard deviation of transient center of mass was calculated over all traversals during which the cell fired exactly one transient. This was to equally weight the contribution of each traversal. **b,** Place field peak locations did not systematically shift over time for bubblegumward (left, p = 0.49, t-test) or pineward (p = 0.69, t-test) place cells. Shift of place field peak was calculated as a place cell's peak firing location during the first half of the recording minus its peak firing location during the second half of the recording. This suggests that odorant depletion (Fig. 1c) does not lead to error accumulation in the place cell network.

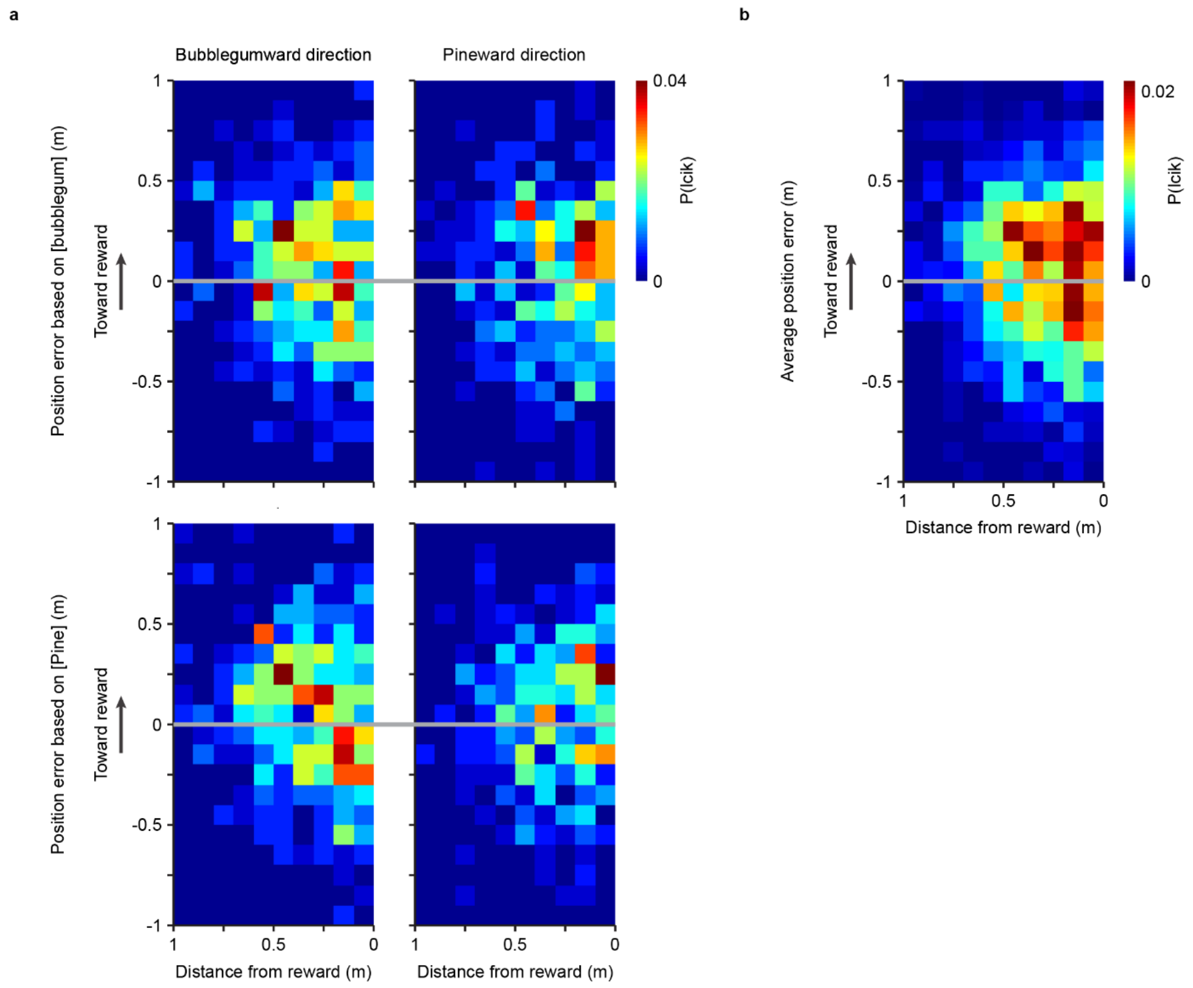

Supplementary Fig. 8: Anticipatory licking in the noisy condition (Fig. 6a) as a function of odor-based position error and distance from reward. a, This is plotted separately for both odors and traversal directions. Licks are pooled across all 5 mice in 8 sessions. Near the reward sites, licking probability appears to be skewed toward odor-based position errors in the direction of the reward. These skews are quantified in the histograms shown in Fig. 6d. **b,** The average of the 4 panels shown in **a**.
